# XBP1 promotes NRAS^G12D^ pre‐B acute lymphoblastic leukaemia through IL‐7 receptor signalling and provides a therapeutic vulnerability for oncogenic RAS

**DOI:** 10.1111/jcmm.17904

**Published:** 2023-09-27

**Authors:** Azam Salimi, Mirle Schemionek‐Reinders, Michael Huber, Margherita Vieri, John B. Patterson, Julia Alten, Tim H. Brümmendorf, Behzad Kharabi Masouleh, Iris Appelmann

**Affiliations:** ^1^ Department of Hematology, Oncology, Hemostaseology and Stem Cell Transplantation, Medical Faculty RWTH Aachen University Aachen Germany; ^2^ Department of Preclinical Imaging and Radiopharmacy, Werner Siemens Imaging Center Eberhard Karls University Tübingen Tübingen Germany; ^3^ Cluster of Excellence iFIT (EXC 2180) "Image Guided and Functionally Instructed Tumor Therapies" Tübingen Germany; ^4^ Medical Faculty, Institute of Biochemistry and Molecular Immunology RWTH Aachen University Aachen Germany; ^5^ Orinove Inc. Newbury Park California USA; ^6^ Department of Pediatrics University Medical Centre Schleswig‐Holstein Kiel Germany

**Keywords:** acute lymphoblastic leukaemia, IL‐7, NRAS^G12D^, PI3K/mTOR, XBP1

## Abstract

Activating point mutations of the RAS gene act as driver mutations for a subset of precursor‐B cell acute lymphoblastic leukaemias (pre‐B ALL) and represent an ambitious target for therapeutic approaches. The X box‐binding protein 1 (XBP1), a key regulator of the unfolded protein response (UPR), is critical for pre‐B ALL cell survival, and high expression of XBP1 confers poor prognosis in ALL patients. However, the mechanism of XBP1 activation has not yet been elucidated in RAS mutated pre‐B ALL. Here, we demonstrate that XBP1 acts as a downstream linchpin of the IL‐7 receptor signalling pathway and that pharmacological inhibition or genetic ablation of XBP1 selectively abrogates IL‐7 receptor signalling via inhibition of its downstream effectors, JAK1 and STAT5. We show that XBP1 supports malignant cell growth of pre‐B NRAS^G12D^ ALL cells and that genetic loss of XBP1 consequently leads to cell cycle arrest and apoptosis. Our findings reveal that active XBP1 prevents the cytotoxic effects of a dual PI3K/mTOR pathway inhibitor (BEZ235) in pre‐B NRAS^G12D^ ALL cells. This implies targeting XBP1 in combination with BEZ235 as a promising new targeted strategy against the oncogenic RAS in NRAS^G12D^‐mutated pre‐B ALL.

## 1 INTRODUCTION

Precursor‐B cell acute lymphoblastic leukaemia (pre‐B ALL) is often driven by genetic alterations including either activating point mutations, chromosomal reciprocal translocations such as the Philadelphia chromosome t(9;22) or aberrant expression of B cell‐specific transcription factors, for example paired box protein 5 (PAX5) and IKAROS family zinc finger 1 (IKZF1).^1^, ^2^ Point mutations in RAS and its downstream effectors act as driver mutations in T‐ALL. However, in B‐ALL, activating *RAS* mutations, namely in *NRAS* and *KRAS*, are frequently found upon relapse and are known to confer resistance to chemotherapy^1^, ^3^, ^4^, ^5^ by aberrant activation of the downstream signalling cascade via RAF/MEK/ERK with a connection to PI3K/AKT/mTOR signalling.^6^, ^7^, ^8^ The prevalence of RAS pathway mutations has been found to differ significantly depending on the selection of the study population and varies from around 10% in a study with no preselection of the study population to 50% in a population encompassing high‐risk common ALL.^7^, ^9^, ^10^, ^11^ Activation of RAS–ERK signalling results in the expression of MAPK negative regulators dual specificity phosphatase 6 (DUSP6), ETS variant 5 (ETV5) and sprouty RTK signalling antagonist 2 (SPRY2), which regulate RAS–ERK signalling via a negative feedback loop.^12^, ^13^, ^14^, ^15^ Interleukin‐7 (IL‐7) is a critical cytokine for survival, homeostasis and development of B and T cells.^16^, ^17^, ^18^ Aberrant IL‐7RA signalling contributes to malignant transformation of pre‐B ALL through the activation of the downstream signalling elements JAK1‐STAT5 and PI3K‐mTOR.^17^, ^18^, ^19^ Indeed, PI3K is a downstream target of both the IL‐7R and the RAS signalling pathways and plays a crucial role in the development of pre‐B ALL.^17^, ^20^, ^21^ Recent studies showed that the unfolded protein response (UPR) network and particularly its IRE1α‐XBP1 axis is activated in a variety of solid tumours and hematologic malignancies.^22^, ^23^ The UPR mediates its function via three signalling branches at the membrane of the endoplasmic reticulum (ER). The most conserved branch is the inositol‐requiring enzyme 1α (IRE1α, ERN1) signalling pathway which is activated to alleviate ER stress.^24^ The basic leucine zipper transcription factor, X box‐binding protein 1 (XBP1), as the key regulator of the UPR is invoked through unconventional mRNA splicing. Consecutively, the endoribonuclease domain of IRE1α mediates excision of a 26‐nucleotide intron from XBP1 mRNA, leading to a shift in the open reading frame and consequently to the production of a longer isoform of XBP1 referred to as spliced XBP1 (XBP1s). XBP1s, then a more stable protein than XBP1, and an active transcription factor, translocates into the nucleus to regulate the expression of downstream target genes involved in protein homeostasis.^24^, ^25^ It is worth noting that pharmacological targeting of the IRE1α‐XBP1 axis has been shown to be an efficient therapeutic strategy for various cancers, for example multiple myeloma and breast cancer.^22^, ^23^ The small molecule IRE1α RNase inhibitor MKC‐8866 is a member of the hydroxyl aryl aldehydes (HAA) family that selectively targets the IRE1α RNase domain, thereby inhibiting XBP1 splicing.^26^, ^27^ In this study, we reveal the molecular mechanism underlying the oncogenic effects of XBP1 in RAS‐mutated pre‐B ALL. These findings provide not only a better insight into the complexity of RAS signalling in pre‐B ALL but also implicate a novel vulnerability in RAS‐mutated pre‐B ALL targeting the oncogenic RAS.

## 2 METHODS

### 2.1 Mice

Bone marrow cells from murine *Xbp1* ^fl/fl^ ^28^ were isolated and cultured as described in Data S1. All animal experiments were approved by the local authorities of North Rhine‐Westphalia under animal protocol number AZ 84‐02.04.2015.A328.

### 2.2 Primary patient samples

Primary bone marrow (BM) cells from patients with RAS‐Mutated pre‐B ALL were kindly provided by the German Society for Pediatric Oncology and Hematology and the Department of Pediatric Hematology and Oncology, University Hospital Schleswig‐Holstein, Kiel, Germany, concordant with the local ethics committee after written informed consent (Table S8). Healthy Human BM samples were obtained from hip replacement surgeries concordant with the local ethics committee after written informed consent.

### 2.3 Flow cytometry

Cell viability, apoptosis, cell cycle and cellular immunophenotyping were analysed by flow cytometry using the Accuri C6 flow cytometer (BD Bioscience®). The antibodies used in this study are provided in Data S1.

### 2.4 Western blotting

Western blot was described in details in Data S1. The antibodies used in this study are listed in Data S1.

### 2.5 Statistical analysis

Data were analysed using the GraphPad Prism® software. Statistical significances were measured for two conditions by unpaired, two‐tailed, Student's *t*‐test and more than three conditions with Bonferrroni's one‐way analysis of variance (anova). *p* values less than 0.05 were considered statistically significant for all of the analyses, defined as *p < 0.05, **p ≤ 0. 01, ***p ≤ 0.001. The Bliss formula and Chou–Talalay method were employed to examine the potency of combined treatment compared with single treatment.^29^

Additional methods are described in Data S1.

## 3 RESULTS

### 3.1 Targeting NRAS^G12D^ ‐driven *Xbp1s* expression impairs pre‐B ALL cell survival

**FIGURE 1:**
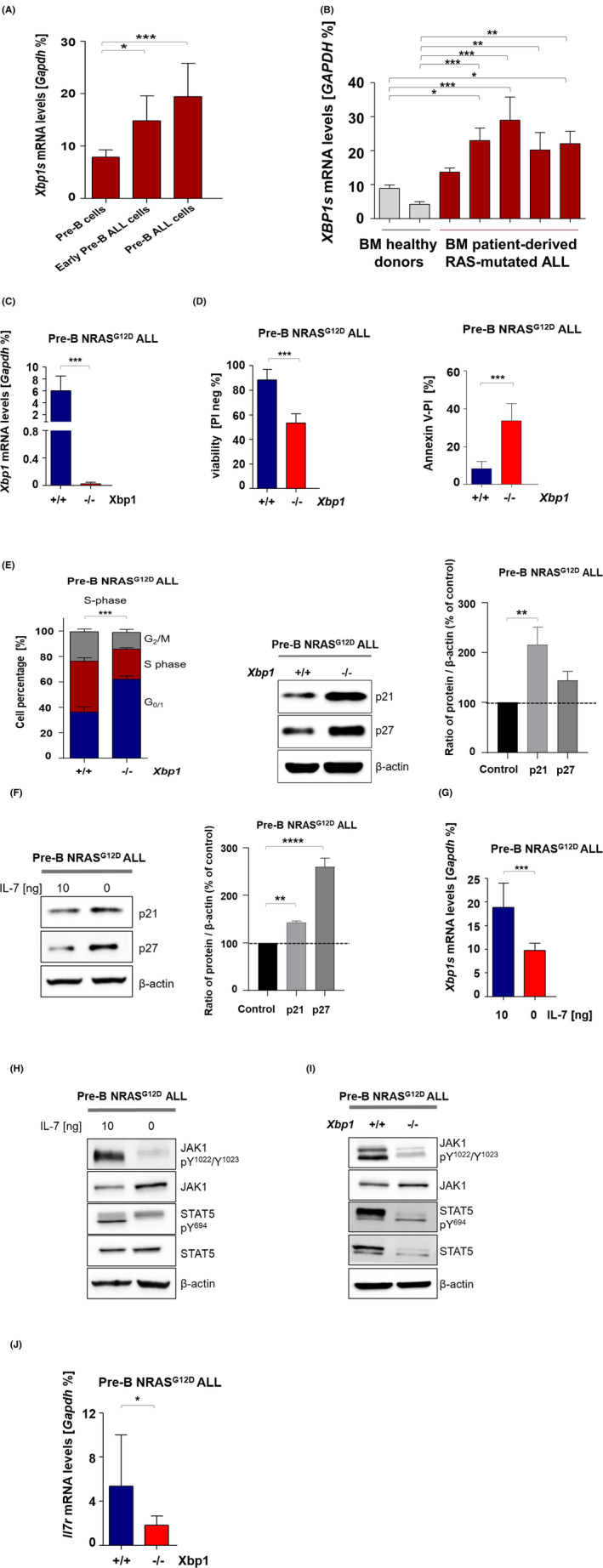
Genetic loss of *Xbp1* induces apoptosis and arrests the cell cycle at G_1_ in pre‐B NRAS^G12D^ ALL cells. (A) Murine bone marrow pre‐B cells were transduced with inducible retroviral NRAS^G12D^. *Xbp1s* mRNA levels were measured in non‐malignant pre‐B cells compared to early pre‐B NRAS^G12D^ ALL cells and late pre‐B NRAS^G12D^ ALL cells using RT‐qPCR. (B) *XBP1s* mRNA levels were measured in RAS mutated ALL from BMMCs of patients (*n* = 5) compared with BM cells from healthy donors (*n* = 2) by RT‐qPCR. P values were calculated by one‐way analysis of variance (anova; *n* = 3. (C) *Xbp1* mRNA levels were measured in *Mx1‐Cre; Xbp1* ^*fl/fl*^ NRAS^G12D^ ALL cells by RT‐qPCR, *n* = 3. (D) Cell viability and apoptotic fraction were analysed in *Mx1‐Cre; Xbp1* ^*fl/fl*^ NRAS^G12D^ ALL cells using PI staining and Annexin‐V/PI staining, respectively, *n* = 3. (E) Cell cycle progression was measured in *Mx1‐Cre; Xbp1* ^*fl/fl*^ NRAS^G12D^ ALL cells using PI staining and protein levels of negative cell cycle regulators p21^CIP1/WAF1^ and p27^KIP1^ as well as beta‐actin as a loading control were assessed by western blot, *n* = 3. The protein levels expressed in relative band density was measured by densitometry analysis. The values represent mean ± SD of three independent experiments. (F) Pre‐B NRAS^G12D^ ALL cells were divided into two subsets. The first subset of cells was cultured in the presence of IL‐7, the second was deprived of IL‐7. Western blot for protein levels of negative cell cycle regulators p21^CIP1/WAF1^ and p27^KIP1^ and beta‐actin as a loading control, *n* = 3. The protein levels expressed in relative band density was measured by densitometry analysis. The values represent mean ± SD of three independent experiments. (G) *Xbp1s* mRNA levels were measured by RT‐qPCR, *n* = 3. *p* values were calculated by Student's *t*‐test. (H, I) Phosphorylation levels of JAK1‐Y^1022^/Y^1023^, STAT5‐Y^694^ and protein levels of JAK1, STAT5 and beta‐actin were assessed by western blot in *Mx1‐Cre; Xbp1* ^*fl/fl*^ NRAS^G12D^ ALL cells and Pre‐B NRAS^G12D^ ALL cells as described above, *n* = 3. (J) *Il7r* mRNA levels were measured in *Mx1‐Cre; Xbp1* ^*fl/fl*^ NRAS^G12D^ ALL cells by RT‐qPCR, *n* = 3.

The significance of XBP1 for the survival and proliferation of pre‐B ALL cells has been previously reported.^30^, ^31^ To further investigate the influence of XBP1 on the progression of RAS‐mediated ALL, we employed a TET‐ON doxycycline (dox)‐induced vector system (Figure S5). For this purpose, bone marrow‐isolated cells were cultured in presence IL‐7 to develop pre‐B cells. We consequently verified pre‐B cell phenotype using B cell lineage cell surface markers, CD19, B220 along with IgM, granulocyte marker Gr‐1 and macrophage marker CD11b. Furthermore, IL‐7‐dependent murine bone marrow pre‐B cells (CD19^+^, B220^+^, IgM^−^, Gr‐1^−^ and CD11b^−^) were retrovirally transduced to express a TET‐ON inducible NRAS^G12D^ mutant in order to regulate oncogenic NRAS^G12D^ expression via dox treatment. We examined the expression signature of *Xbp1s* at different stages of RAS activation: nonmalignant pre‐B cells, early NRAS^G12D^ transduced ALL cells (early pre‐B ALL) and late pre‐B NRAS^G12D^ ALL cells. We observed first that pre‐B NRAS^G12D^ ALL cells show viability around 60% and viability was increased to 90% in response to IL‐7. Furthermore, we named as ‘early NRAS G12D transduced ALL cells’ to define pre‐B NRAS^G12D^ ALL cells 1–3 weeks after marker colony selection. For ‘late pre‐B NRAS G12D ALL cells’, we consider 4–5 weeks after marker colony selection. As expected, *Xbp1s* expression increased upon induction of NRAS^G12D^ in early NRAS^G12D^ transduced ALL cells compared to nonmalignant pre‐B cells. In addition, *Xbp1s* expression was further upregulated in late pre‐B NRAS^G12D^ ALL cells (Figure 1A). In a second approach, we measured expression of *XBP1s* mRNA levels in five RAS‐mutated ALL patients at the time of diagnosis. Quantitative real‐time PCR revealed high expression of *XBP1s* mRNA levels in RAS‐mutated ALL patients compared with healthy donors (Figure 1B) suggesting that XBP1 might be important for in RAS‐driven transformation of pre‐B cells.^30^ To evaluate the functional significance of XBP1 in RAS‐driven transformation of pre‐B cells, we utilized a conditional *Xbp1* knockout mouse model. In this model, IL‐7‐dependent murine bone marrow *Mx1‐Cre; Xbp1* ^*f/f*^ pre‐B cells (CD19^+^, B220^+^, IgM^−^, Gr‐1^−^ and CD11b^−^) were transduced with a TET‐ON inducible NRAS^G12D^ retrovirus. MX1‐Cre expression was induced by interferon α (IFN‐α) treatment, which mediated excision of *Xbp1* (Figure S1A). The mRNA expression levels of *Xbp1* in *Mx1‐Cre; Xbp1* ^*fl/fl*^ pre‐B NRAS^G12D^ ALL cells were analysed, confirming a deletion of *Xbp1* at the genetic level (Figure 1C).

Genetic loss of *Xbp1* induced apoptosis in pre‐B NRAS^G12D^ ALL cells. This is in accordance with previous findings indicating a pivotal role of XBP1 for pre‐B ALL survival^32^, ^33^ (Figures 1D and S1B). Moreover, genetic ablation of *Xbp1* caused cell cycle arrest in the G_1_ phase and was accompanied by an upregulation of cyclin‐dependent kinase inhibitors p21^CIP1/WAF1^ and p27^KIP1^ (Figure 1E), implicating XBP1 as critical for the survival and proliferation of the pre‐B NRAS^G12D^ ALL subset. We consequently examined whether genetic loss of *Xbp1* could alter the malignant pre‐B cell phenotype in *Mx1‐Cre; Xbp1* ^*f/f*^ pre‐B NRAS^G12D^ ALL cells. Our analyses resulted similar phenotype in the presence or absence of XBP1 (Figure S1C).

### 3.2 XBP1 acts as a downstream linchpin of the IL‐7 receptor signalling pathway

As delineated above, IL‐7 is pivotal for survival and proliferation of pre‐B NRAS^G12D^ ALL cells. To further validate this observation, we supplemented a subset of pre‐B NRAS^G12D^ ALL cells with 10 ng/mL IL‐7 and starved a second subset of IL‐7 for 24 h. Cell viability significantly decreased in the IL‐7‐deprived cells compared with IL‐7‐stimulated pre‐B NRAS^G12D^ ALL cells. Furthermore, we showed that XBP1 is pivotal for survival of pre‐B ALL cells and addition of IL‐7 did not revers cell death mediated by genetic loss of *Xbp1* (Figure S2A). For a more in‐depth analysis of the effect of IL‐7 on proliferation of pre‐B NRAS^G12D^ ALL cells, we measured protein levels of the G_1_ phase negative cell cycle regulators p21^CIP1/WAF1^ and p27^KIP1^ as described above. Cells in the absence of IL‐7 increased the expression of p21 and p27, implicating a crucial role of IL‐7 in cell cycle progression of pre‐B NRAS^G12D^ ALL cells (Figure 1F). Next, we analysed the molecular signature of *Xbp1s* expression in response to IL‐7 stimulation. Interestingly, we observed a downregulation of *Xbp1s* expression levels in IL‐7‐deprived cells compared with IL‐7‐stimulated cells (Figure 1G). As a consequence of the observed increase of Xbp1 expression upon IL‐7R activation (Figure 1G), we next examined whether XBP1 was relevant for IL‐7 receptor signalling and analysed the phosphorylation of IL‐7 receptor downstream effectors JAK1 and STAT5 in the absence of XBP1. Indeed, genetic loss of *Xbp1* substantially reduced phosphorylation levels of JAK1‐Y^1022^/Y^1023^ and STAT5‐Y^694^ even when supplemented with IL‐7 (Figure 1H). In addition, expression levels of *Il7ra* were significantly reduced in the absence of XBP1 (Figure 1I). Previous studies revealed that STAT5 positively regulates *Xbp1* expression in the nucleus.^30^, ^32^, ^34^ Here, we showed that inhibition of STAT5 occurs as a consequence of a negative feedback regulation upon genetic loss of *Xbp1* in pre‐B NRAS^G12D^ ALL cells. Similar to our findings after genetic deletion of *Xbp1*, the withdrawal of IL‐7 for 24 h abolished JAK1 and STAT5 phosphorylation in comparison with IL‐7‐stimulated cells (Figure 1J). These results demonstrate that XBP1 significantly contributes to IL‐7 receptor signalling and loss of XBP1 in turn abrogates IL‐7 signalling via the inhibition of the JAK1‐STAT5 axis. In addition and supporting, suppression of IL‐7 receptor signalling and additive genetic loss of *Xbp1* resulted in increased phosphorylation of P38‐T^180^/Y^182^ and JNK‐T^183^/Y^185^ in pre‐B NRAS^G12D^ ALL cells, leading to cell death mediated by activation of stress response kinases (Figure S2B).

### 3.3 Active XBP1 decreases RAS signalling strength to maintain homeostasis of pre‐B NRAS^G12D^ ALL cells

**FIGURE 2:**
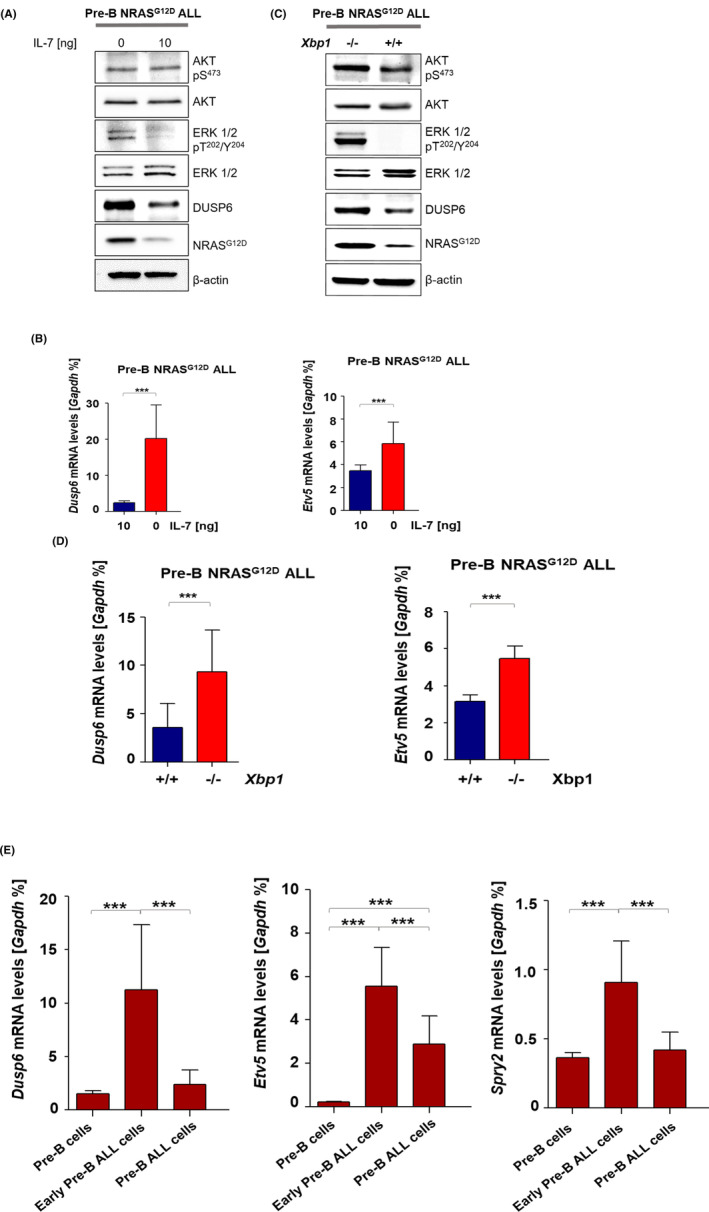
XBP1 Modulates the RAS signalling strength in pre‐B NRAS^G12D^ ALL cells. (A) Pre‐B NRAS^G12D^ ALL cells were cultured as described in Figure 1. Total protein levels of NRAS^G12D^, DUSP6, AKT, ERK1/2 and beta‐actin as a loading control along with phosphorylated levels of ERK‐T^202^/Y^204^ and AKT‐S^473^ were measured by western blot, *n* = 3. (B) *Dusp6* and *Etv5* mRNA levels were examined by RT‐qPCR as mentioned above, *n* = 3. (C) Western blot analysis on *Mx1‐Cre; Xbp1* ^*fl/fl*^ NRAS^G12D^ ALL cells for phosphorylation levels of ERK‐T^202^/Y^204^, AKT‐S^473^ and protein levels of NRAS^G12D^, DUSP6, AKT, ERK1/2, beta‐actin, *n* = 3. (D) *Dusp6* and *Etv5* mRNA levels in in *Mx1‐Cre; Xbp1* ^*fl/fl*^ NRAS^G12D^ ALL cells using RT‐qPCR, *n* = 3. Significance value *p* was again calculated by Student's *t*‐test. (E) MAPK‐negative regulators, *Dusp6, Etv5* and *Spry2* mRNA levels were studied as explained in Figure 1 using RT‐qPCR, *n* = 3. *p* values were calculated by one‐way analysis of variance (anova).

To investigate the capacity of IL‐7 signalling and XBP1 to counteract the RAS signalling pathway, we examined whether removal of IL‐7 functionally impairs RAS signalling via IL‐7 receptor signalling in pre‐B ALL cells. For this purpose, we measured expression levels of RAS downstream targets including ERK1/2 and MAPK‐negative regulators, DUSP6 and ETV5 on the mRNA and the protein level after 24 h of depletion of IL‐7. We observed that cells without IL‐7 strongly induced DUSP6 and ETV5 at both the mRNA and the protein level (Figure 2A,B). The high phosphorylation level of ERK‐T^202^/Y^204^ along with an increased expression of the NRAS^G12D^ protein demonstrates active RAS in IL‐7 nonstimulated cells (Figure 2A,B). A similar expression pattern resulted from genetic loss of *Xbp1* in pre‐B ALL cells. Pre‐B NRAS^G12D^ ALL cells lacking XBP1 augmented phosphorylation of ERK‐T^202^/Y^204^ and AKT‐S^473^ and also induced the expression of DUSP6 and NRAS^G12D^ at the mRNA and protein level (Figure 2C,D). These findings indicate that targeting the IL‐7 receptor signalling as well as genetic loss of *Xbp1* activates the Ras–Erk signalling pathway in pre‐B NRAS^G12D^ ALL cells. Next, we determined the RAS pathway activation signature during pre‐B NRAS^G12D^ ALL progression by measuring expression levels of *Dusp6*, *Etv5* and *Spry2*, all are known to be expressed in response to ERK activation. We assessed mRNA levels of these MAPK‐negative regulator genes at three stages of pre‐B NRAS^G12D^ ALL progression (pre‐B cell, early NRAS^G12D^ transduced cells and late pre‐B NRAS^G12D^ ALL cells) and observed that the expression levels of *Dusp6*, *Etv5* and *Spry2* increased upon induction of NRAS^G12D^ in early NRAS^G12D^ transduced cells compared with pre‐B cells (Figure 2E). However, a substantial reduction at the mRNA level of these MAPK‐negative regulator genes was seen in late pre‐B NRAS^G12D^ ALL cells (Figure 2E) suggesting that an acute activation of RAS signalling at the early NRAS^G12D^ transduced stage is later attenuated during disease progression of pre‐B ALL.

### 3.4 Dual PI3K/mTOR inhibitor in the absence of active XBP1 synergistically induces apoptosis in NRAS^G12D^ pre‐B ALL cells

**FIGURE 3:**
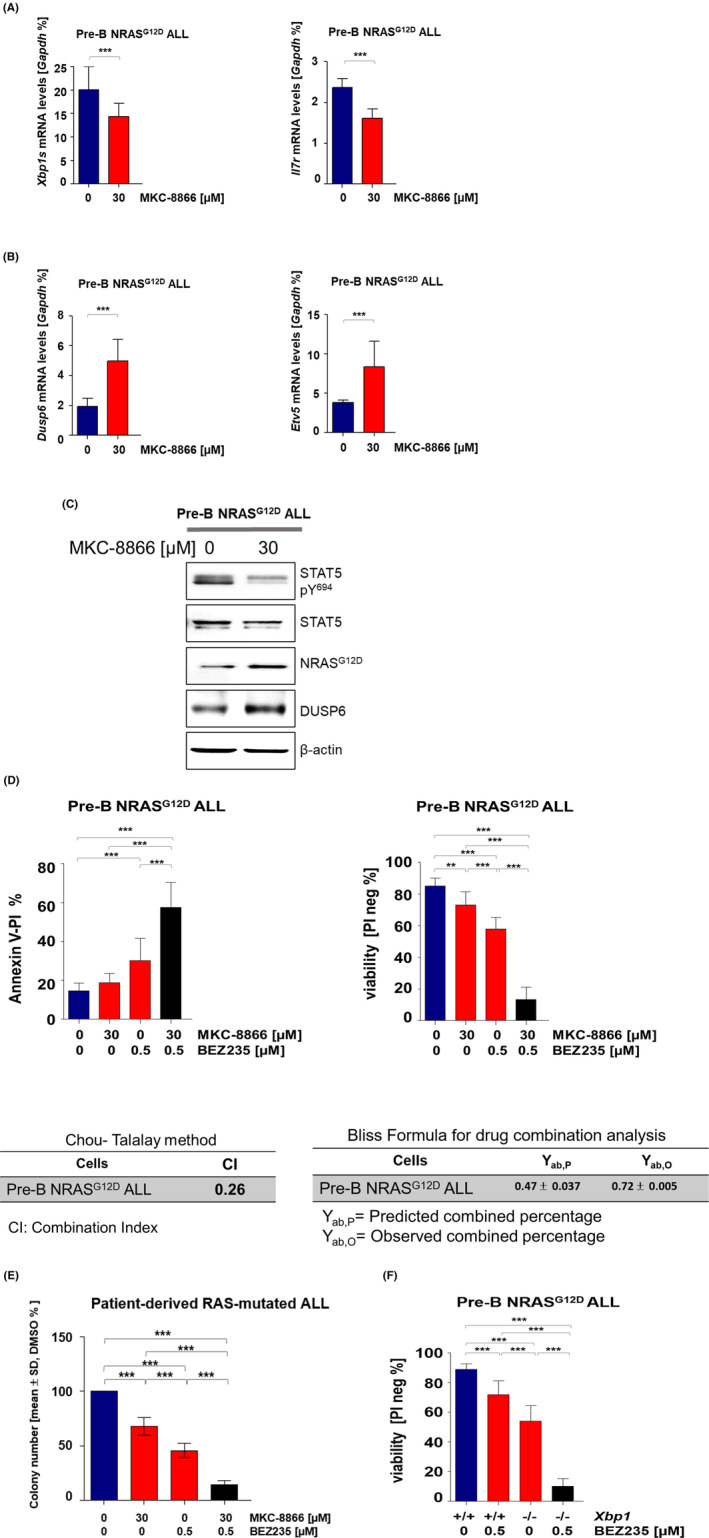
Pre‐B NRAS^G12D^ ALL cells strongly respond to BEZ235 treatment in the absence of active XBP1. Pre‐B NRAS^G12D^ ALL cells were treated with 30 μM of the IRE1α inhibitor MKC‐8866 alone or combined with 0.5 μM of the PI3K/mTOR inhibitor BEZ235. (A, B) *Xbp1*, *Il7r, Dusp6* and *Etv5* mRNA levels were analysed after 24 h treatment with MKC‐8866 by RT‐qPCR, *n* = 3. *p* value was calculated by *t*‐test analysis. (C) Protein levels for NRAS^G12D^, DUSP6, STAT5, beta‐actin as loading control and phosphorylated STAT5‐Y^694^ were measured by western blot, *n* = 3. (D) Cell viability and apoptosis were examined after 5 days of treatment using PI staining and Annexin‐V/PI, respectively; *n* = 3. The table represents synergistic interaction of combined drugs. The percentage of inhibitory effect of combined drugs was calculated by bliss formula. In addition, combination of Index was calculated based on Chou‐Talalay. (E) Cell viability of *Mx1‐Cre; Xbp1* ^*fl/fl*^ NRAS^G12D^ ALL cells after 72 h of treatment with 0.5 μM BEZ235 by PI staining, *n* = 3. (F) Primary BMMCs RAS‐mutated ALL were treated with MKC‐8866 30 μM or BEZ235 0.5 μM and combination of both drugs, *n* = 5. CFU assay was performed using 10.000 cells and were counted after 14 days. Error bars represent mean ± SD for each group. *p* values were calculated using one‐way anova.

To determine the therapeutic potential of the selective IRE1α small molecule inhibitor MKC‐8866 that targets specifically the RNase domain of the IRE1α protein, we first analysed the effect of MKC‐8866 treatment on *Xbp1* splicing in pre‐B ALL cells. As expected, treatment of pre‐B ALL cells with MKC‐8866 for 24 h significantly reduced the expression of *Xbp1* spliced variant, *Xbp1s* (Figure 3A). In addition, treated cells reduced expression levels of *Il7r*, indicating pharmacological inhibition of IRE1α negatively impacting IL‐7R signalling through the loss of XBP1 splicing in pre‐B ALL cells (Figure 3A). However, MKC‐8866 showed minor adverse effects on the viability of pre‐B NRAS^G12D^ ALL cells (Figure S3D). As a next step, we compared the efficacy of MKC‐8866 to the genetic loss of *Xbp1* by measuring phosphorylation levels of STAT5‐Y^694^ along with expression levels of NRAS^G12D^ and DUSP6 at both the mRNA and the protein level (Figure 3B,C) and indeed observed comparable effects.

Next, the selective impact of MKC‐8866 on IL‐7R signalling inhibition and concurrent activation of RAS led us to investigate the potential of targeting the RAS signalling pathway by applying the MEK inhibitor PD0325901 in combination with MKC‐8866 in our NRAS^G12D^ pre‐B ALL cells. In contrast to our predictions, the viability of cells treated with PD0325901 in combination with MKC‐8866 was only minimally reduced in comparison with single treatments as assessed by PI staining (Figure S3E). We also detected comparable effects in cells after genetic loss of *Xbp1* when PD0325901 treatment showed an insubstantial effect on the reduction of cell viability (Figure S3E). As a second approach to indirectly target RAS‐mediated signalling in NRAS^G12D^ pre‐B ALL, we investigated PI3K signalling, which, when activated, is strongly correlated with tumour progression in hematologic malignancies.^33^, ^35^, ^36^ Therefore, we analysed the efficiency of PI3K inhibition by applying the dual PI3K/mTOR inhibitor BEZ235 on pre‐B ALL cells together with MKC‐8866 and observed a striking increase in the fraction of apoptotic pre‐B ALL cells (Figures 3D and S3B,D). Bliss formula calculation revealed that BEZ235 in combination with MKC‐8866 synergistically reduces the viability of RAS‐mutated pre‐B ALL cells (Figure 3D). To support this finding, we measured the colony forming capacity upon the above‐mentioned treatment strategy. Colony number was notably reduced upon combined treatment compared with single treatment or untreated cells (Figure S3A). In a similar way, we tested the efficacy of BEZ235 together with genetic ablation of *Xbp1*. As shown in Figure 3F, addition of BEZ235 strongly induced cell death in the absence of XBP1, implying that the genetic ablation of *Xbp1* or its pharmacological inhibition with MKC‐8866 strongly sensitizes pre‐B ALL cells to dual inhibition of PI3K/mTOR. To validate our results, colony forming ability was determined in RAS‐mutated ALL cases under our combined treatment strategy. Colony formation after combination therapy was significantly reduced in comparison of untreated cells and single treatment with MKC‐8866 or BEZ235 (Figure 3E), confirming the efficacy of our combined drugs against RAS‐driven pre‐B ALL.

### 3.5 Inhibition of PI3K/mTOR signalling in the absence of active XBP1 repeals IL‐7 signalling and induces senescence

**FIGURE 4:**
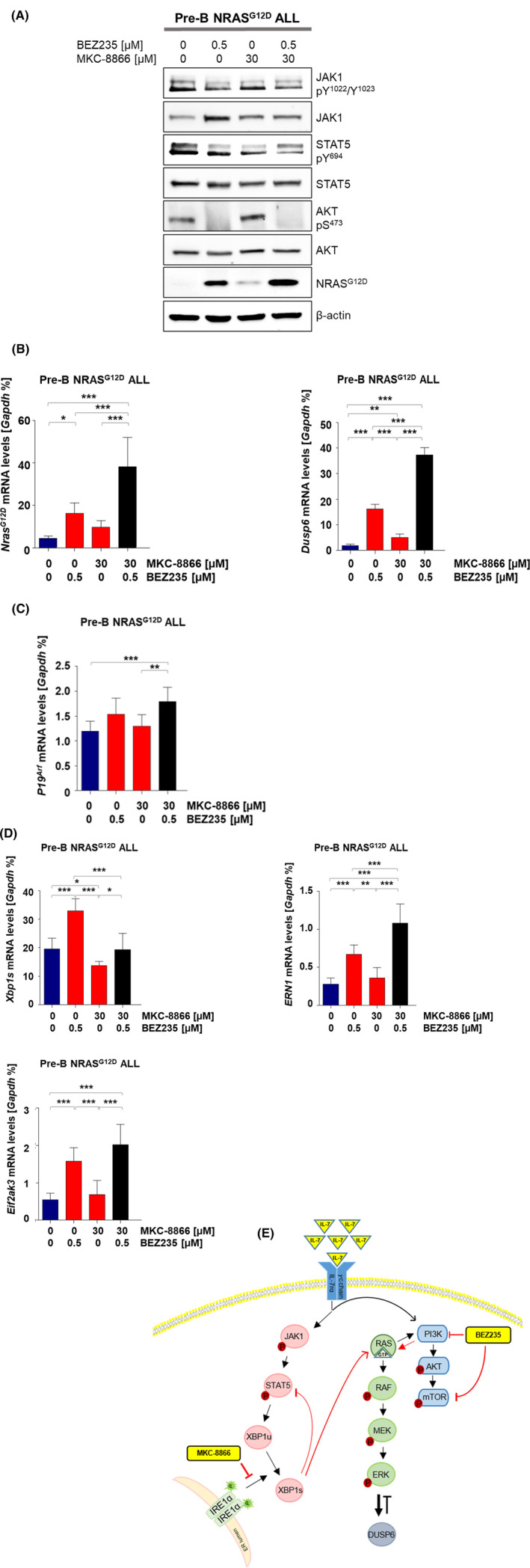
Inhibition of PI3K in the absence of active XBP1 causes acute hyper‐activation of Ras–Erk signalling. Pre‐B NRAS^G12D^ ALL cells were treated as described in Figure 3. (A) Western blot analysis for NRAS^G12D^, JAK1, STAT5, AKT and beta‐actin as loading control along with phosphorylated JAK1‐Y^1022^/Y^1023^, STAT5‐Y^694^ and AKT‐S^473^ after 16‐h treatment, *n* = 3. (B–D) *Nras* ^*G12D*^, *Dusp6*, *p19* ^*Arf*^, *Xbp1s*, *ERN1* and *Eif2ak3* mRNA levels were analysed by RT‐qPCR after 16‐h treatment, *n* = 3. The *p* value was calculated by one‐way analysis of variance (anova). (E) A schematic overview of interplay between PI3K/mTOR signalling and IRE1α‐XBP1 in pre‐B NRAS^G12D^ ALL cells.

Finally, to unveil the mechanism underlying cell death mediated by our combined regimen, we analysed expression pattern changes resulting from single and dual treatment at both the mRNA and the protein level. Dual treatment resulted in decreased phosphorylation levels of STAT5‐Y^694^ and JAK1‐Y^1022^/Y^1023^ (Figure 4A) compared to single treatment. As expected, BEZ235 as a single and dual treatment abolished phosphorylation of AKT‐S^473^ in pre‐B ALL cells (Figure 4A). Interestingly, targeting PI3K/mTOR signalling along with MKC‐8866 strongly induced expression of NRAS^G12D^ and its downstream target *Dusp6* (Figure 4A,B). BEZ235 treatment upon loss of *Xbp1* exhibited a similar pattern on expression of both RAS and IL‐7R signalling downstream targets (Figure S4A,B). Accordingly, gene expression analysis indicated that BEZ235 in combination with MKC‐8866 fully blocks IL‐7 signalling and causes an aberrant activation of NRAS^G12D^ in pre‐B ALL cells. In addition, it has been shown that targeting Xbp1 induce cell senescence in pre‐B ALL cells.^30^ we tested if hyperactive RAS signalling resulting from dual treatment can synergistically increase cell senescence. We therefore measured expression levels of *p19* ^*Arf*^ as a hallmark of senescence.^37^, ^38^ However, we observed relatively minor upregulation of p19arf (Figures 4C and S4D).

We also detected elevated levels of the UPR genes *Ern1* and *Eif2ak3* following single and dual treatment showing that UPR‐associated genes are upregulated following RAS activation as an anticipated stress response of the cell (Figures 4D and S4C).

To determine whether the aberrant increase of NRAS^G12D^ expression levels observed in our cells treated with the combination of MKC‐8866 and BEZ235 is mediated through stabilizing NRAS mRNA, we pretreated pre‐B NRAS^G12D^ ALL cells with either MKC‐8866 or BEZ235 as single agents, followed by actinomycin D treatment to inhibit RNA transcription. However, both BEZ235 and MKC‐8866 had an only minor impact on NRAS^G12D^ mRNA stability in pre‐B ALL cells (Figure S4E). Altogether, our findings suggest that IRE1α‐XBP1 signalling is involved in regulation of RAS signalling strength in pre‐B NRAS^G12D^ ALL.

## 4 DISCUSSION

In this study, we uncovered the significance of the UPR and particularly XBP1 in NRAS^G12D^‐driven pre‐B leukemogenesis and identified its potency as a therapeutic target in this context. Our findings shed light on the molecular mechanism underlying XBP1‐mediated survival of pre‐B ALL cells. The UPR network is required for tumour maintenance and progression in a wide range of solid tumours and hematologic malignancies.^24^, ^30^, ^39^, ^40^, ^41^ Indeed, cancer cells are dependent on the UPR to relieve cell stress induced by their high proliferation rate and the likewise high protein demand and perturbed proteostasis. Here, we showed that IRE1α‐XBP1 signalling is strongly increased upon NRAS^G12D^ activation in pre‐B ALL cells. Consecutively, genetic deletion of *Xbp1* arrested cell cycle progression at the G_1_ phase and induced apoptosis in NRAS^G12D^ pre‐B ALL cells. Interestingly, we discovered that XBP1 acts as downstream effector of IL‐7R signalling and thereby promotes proliferation and survival of pre‐B ALL cells. We were able to document a regulation of *Xbp1s* expression by IL‐7R signalling in agreement with current data, particularly ChIP analyses, showing positive effects of STAT5 on *Xbp1* expression.^30^, ^32^, ^34^ Similarly, we observed that targeting of IL‐7R signalling by withdrawal of the IL‐7 cytokine itself led to STAT5 inactivation and decreased expression of *Xbp1s*, ultimately resulting in a reduction of cell viability and cell cycle arrest at the G_1_ phase via induction of cell cycle negative regulators p21^CIP1/WAF1^ and p27^KIP1^. These data show that IL‐7R signalling through its downstream effector XBP1 exerts positive effects on cell survival and proliferation in NRAS^G12D^ pre‐B ALL cells. This is in line with previous findings demonstrating the imperative requirement of IL‐7R signalling for B cell survival.^16^, ^42^

To further elucidate the functional role of XBP1 in IL‐7R signalling, we investigated the effects of genetic loss of *Xbp1*. Upon loss of XBP1, RAS signalling was activated and significantly increased expression levels of NRAS^G12D^ and DUSP6 along with high phosphorylation levels of ERK1/2 and AKT were observed. In the absence of XBP1, our NRAS^G12D^ pre‐B ALL cells showed strongly increased phosphorylation levels of JNK and p38, suggesting that cell death is mediated by activation of these MAPK signalling pathways under cell stress conditions. Our results are in accordance with data from other studies demonstrating that IL‐7 withdrawal induces cell stress and thereby leads to the activation of stress signalling via p38 MAP kinase and c‐Jun‐N‐terminal kinase (JNK) in IL‐7 dependent cells.^43^, ^44^ We further analysed signalling events following inhibition of the IL‐7R by removal of IL‐7 and identified very similar gene expression patterns in comparison with the genetic ablation of *Xbp1*. Pre‐B NRAS^G12D^ ALL cells in the absence of IL‐7 showed high expression levels of NRAS^G12D^, increased phosphorylation of ERK1/2 and a consecutive induction of MAPK negative regulators (DUSP6 and ETV5) along with reduced phosphorylation of the downstream effectors JAK1 and STAT5. These results confirm that IL‐7R signalling drastically impedes RAS signalling via XBP1 as a key mediator.

Surprisingly, we observed that the RAS–ERK signalling was less pronounced in late pre‐B NRAS^G12D^ ALL compared to early NRAS^G12D^ transduced cells. A substantial increase in the expression levels of MAPK‐negative regulators *Dusp6*, *Etv5* and *Spry2* as a consequence of RAS–ERK activation in early NRAS^G12D^ transduced cells was also demonstrated but diminished with progression to late pre‐B NRAS^G12D^ ALL cells. Indeed, lowering the activation of RAS–ERK signalling correlated with an increased cell viability of pre‐B NRAS^G12D^ ALL, suggesting that activation of XBP1 during the establishment of ALL hinders the RAS signalling pathway to ensure the maintenance of homeostasis in pre‐B ALL. Our findings are in accordance with previous studies documenting that hyperactive ERK signalling induces apoptosis^45^, ^46^ and suggest that the IRE1α‐XBP1 signalling axis buffers the intensity of RAS signalling hence contributing to cell survival of pre‐B NRAS^G12D^ ALL cells.

Efficient inhibition of *Xbp1* splicing and consecutive inactivation of XBP1 in pre‐B NRAS^G12D^ ALL cells was induced using MKC‐8866 in this present study. In order to discriminate off‐target effects of high concentrations of MKC‐8866 in blocking pre‐B ALL cells growth, we employed genetic loss of *Xbp1* in parallel, and IRE1 targeting using MKC‐8866 revealed a similar phenotype compared with genetic loss of *Xbp1*. MKC‐8866 treatment reduced cell viability and abrogated IL‐7R signalling to a very similar extent as genetic loss of *Xbp1*. In parallel, we found that MKC‐8866 activates the RAS signalling pathway via increased expression of NRAS^G12D^. Furthermore, we verified the pre‐clinical therapeutic potential of pharmacological IRE1α inhibition with MKC‐8866 on pre‐B ALL cells and consequently analysed the relevance of PI3K/mTOR signalling in pre‐B ALL cells. PI3K/mTOR signalling acts as a pivotal downstream effector of both IL‐7 and RAS signalling mediating cell proliferation.^20^, ^35^, ^47^ We hypothesized that targeting of both IL‐7R and RAS signalling via PI3K/mTOR could provide a promising therapeutic approach in pre‐B ALL cells. Intriguingly, we observed that the dual inhibitor of PI3K/mTOR, BEZ235, in combination with MKC‐8866 synergistically induced cell death in pre‐B ALL cells. In addition to pharmacological inhibition of XBP1, we confirmed a similar effect of BEZ235 upon genetic loss of *Xbp1*, suggesting that an interplay between PI3K/mTOR signalling and IRE1α‐XBP1 promotes survival of pre‐B NRAS^G12D^ ALL cells and that these cells in the absence of active XBP1 are particularly vulnerable to BEZ235 treatment. However, in order to simulate an exact replica of the human ALL disease, one would need to examine the therapeutic value of IRE1α and BEZ235 inhibition in patient derived xenograft (PDX) model with diagnosed or relapsed tumour samples from patients with RAS‐mutated ALL.

To elucidate the molecular mechanism underlying cell death mediated by MKC‐8866 together with BEZ235, we examined the expression levels of several genes involved as downstream targets of both IL‐7R and RAS signalling either upon genetic loss of *Xbp1* or MKC‐8866 treatment in combination with BEZ235. We observed that treatment of pre‐B ALL cells lacking active XBP1 with BEZ235 resulted in aberrant expression of NRAS^G12D^ and its downstream target *Dusp6* while phosphorylation levels of STAT5 and JAK1 strongly decreased. We also detected an increase in the expression levels of *Il7r* upon BEZ235 treatment. This effect was partly graduated by MKC‐8866 treatment. It is worthy of note that *Il7r* expression is regulated by FOXO1.^48^, ^49^, ^50^ FOXO1 is arrested in the cytoplasm through phosphorylation at three sites by the AKT protein kinase. Inhibition of AKT releases FOXO1 into the nucleus and activates expression of target genes, namely *Il7r*.^47^, ^50^, ^51^ In accordance with this concept, we suggest that BEZ235 treatment via inactivation of AKT might lead to nuclear import of FOXO1 and induction of *Il7r* expression. In addition, the high expression of UPR genes, namely *Ern1* and *Eif2ak3*, in response to BEZ235 as well as combined treatment, indicates the activation of ER stress through the induction of the UPR as an adaptive response to shield cells against the cytotoxic effects of our dual treatment. We also noticed that combined treatment induces cell senescence via induction of *p19* ^*Arf*^ in comparison with single BEZ235 treatment, implicating that XBP1 might be required to RAS‐induced senescence in pre‐B NRAS^G12D^ ALL cells. Indeed, this finding is supported by recent data also demonstrating that the aberrant RAS signalling drive cellular senescence in absence of active IRE1α‐XBP1 signalling.^52^, ^53^ Our findings show that BEZ235 together with MKC‐8866 blocks IL‐7R signalling and synergistically increases RAS–ERK signalling activation. Indeed, inhibition of the IRE1α‐XBP1 axis activates PI3K/mTOR signalling as a compensatory pathway to prevent aberrant activation of RAS signalling and therefore protects cells against deleterious effects of hyperactive RAS–ERK signalling (Figure 4E).

Taken together, our data point towards an important crosstalk between IRE1α‐XBP1 and PI3K/mTOR signalling pathways in RAS‐mutated ALL, highlighting the requirement of XBP1 in RAS‐mediated leukemogenesis. Targeting XBP1 as a pivotal effector of the UPR in combination with the dual inhibitor of PI3K/mTOR, BEZ235, could therefore represent a promising potential therapeutic approach for the treatment of oncogenic RAS in NRAS^G12D^ pre‐B ALL.

## AUTHOR CONTRIBUTIONS

**Azam Salimi:** Data curation (lead); formal analysis (lead); investigation (lead); methodology (lead); visualization (lead); writing – original draft (lead). **Mirle Schemionek‐Reinders:** Writing – review and editing (equal). **Michael Huber:** Writing – review and editing (equal). **Margherita Vieri:** Formal analysis (supporting). **John B. Patterson:** Resources (equal). **Julia Alten:** Resources (equal). **Tim H. Brümmendorf:** Writing – review and editing (equal). **Behzad Kharabi Masouleh:** Conceptualization (equal); funding acquisition (equal); writing – review and editing (equal). **Iris Appelmann:** Conceptualization (equal); formal analysis (supporting); funding acquisition (equal); investigation (supporting); methodology (supporting); project administration (lead); supervision (lead); validation (lead); visualization (supporting); writing – original draft (supporting); writing – review and editing (equal).

## CONFLICT OF INTEREST STATEMENT

IA and BKM received funding from Takeda Pharmaceutical®. IA received funding from Novartis®.

## Supporting information


Figure S1.

Figure S2.

Figure S3.

Figure S4.

Figure S4.

Figure S5.

Data S1.

## Data Availability

Data available on request from the authors.
