# Supplementary figures and images for: XBP1 promotes NRASG12D pre‐B acute lymphoblastic leukaemia through IL‐7 receptor signalling and provides a therapeutic vulnerability for oncogenic RAS
Source: J Cell Mol Med. 2023 Sep 27;27(21):3363–77. doi: 10.1111/jcmm.17904 (PMC10623536; doi:10.1111/jcmm.17904)

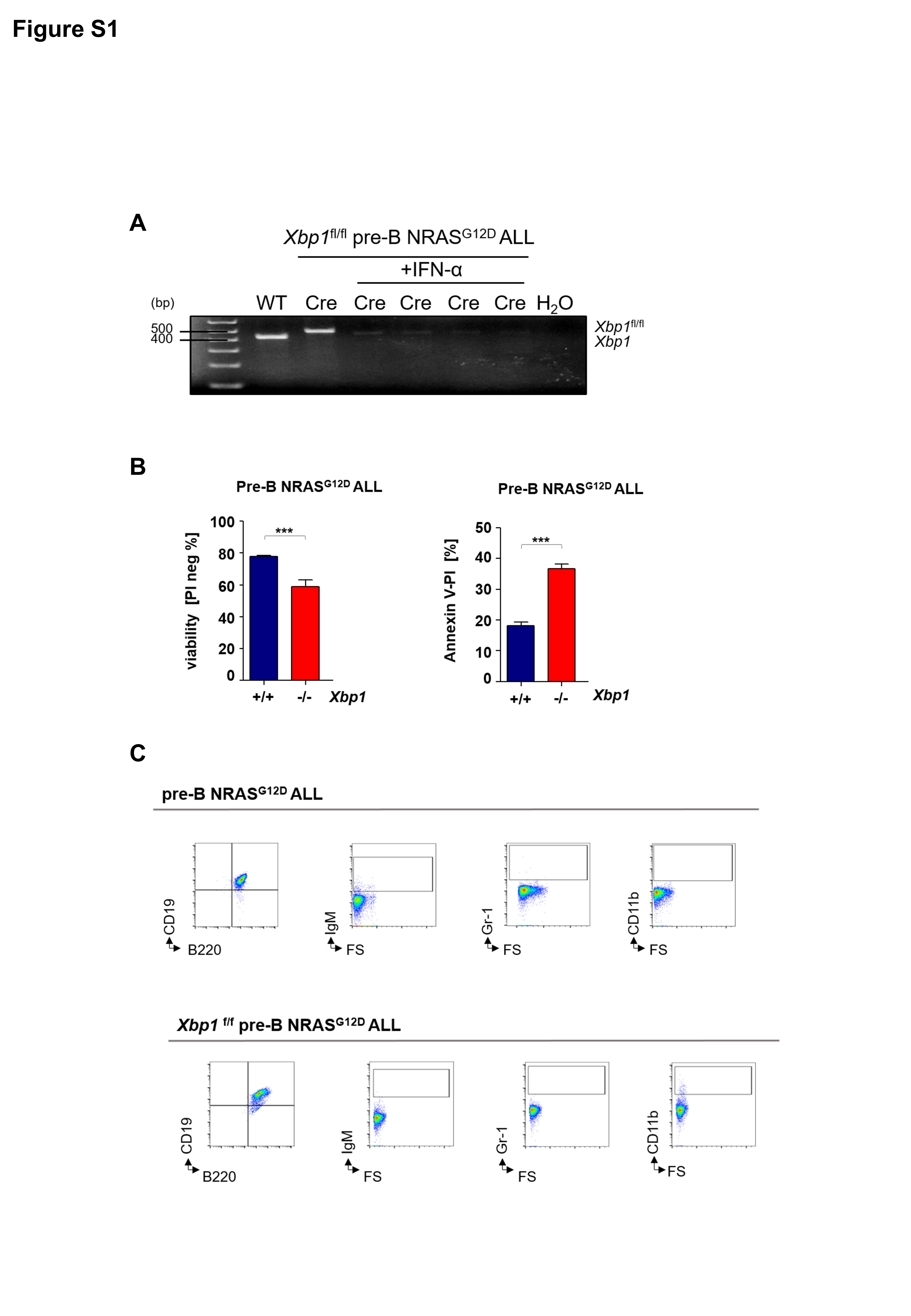

Supplement: Supplementary file 1 — Figure S1. [file JCMM-27-3363-s003.tif]

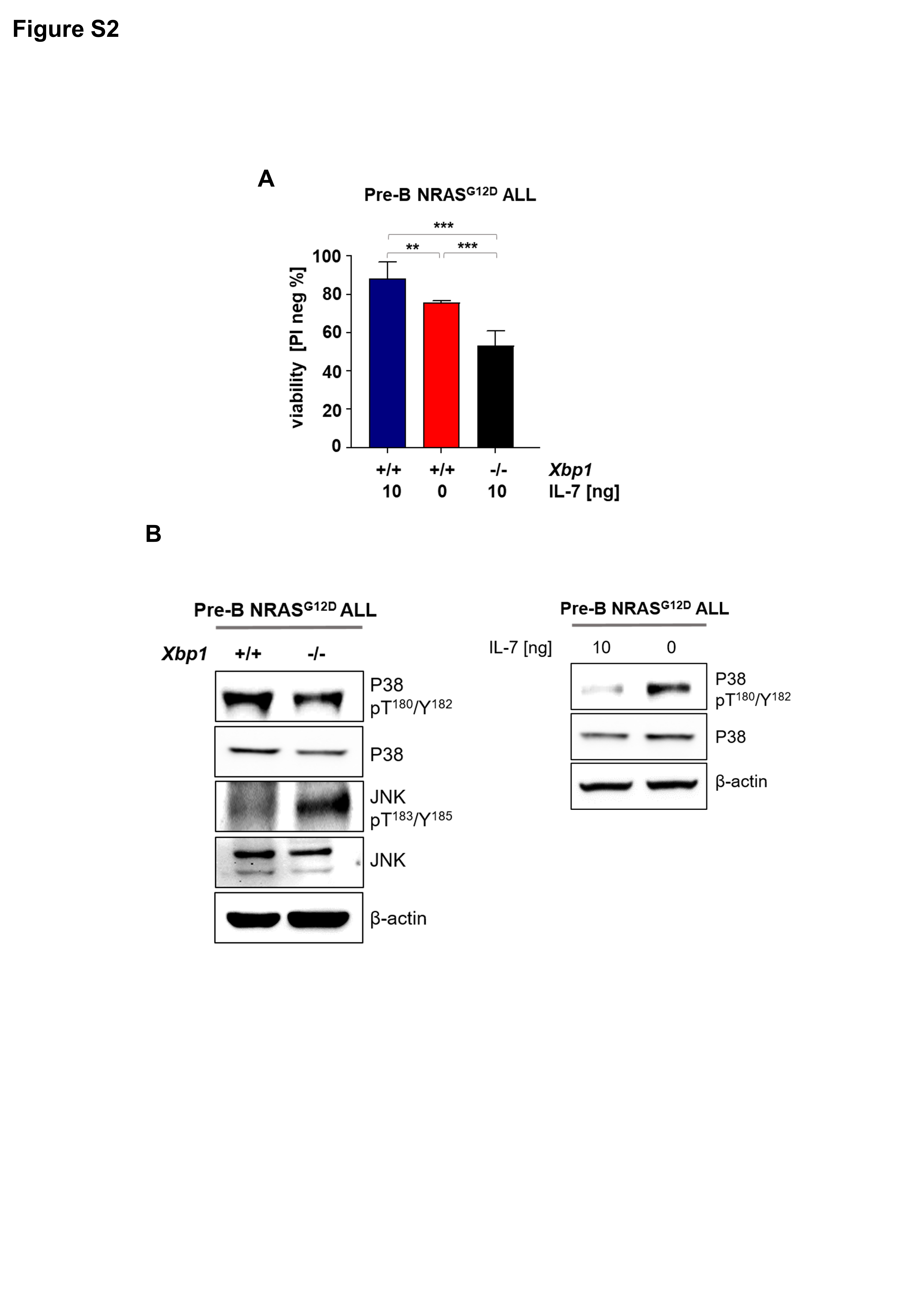

Supplement: Supplementary file 2 — Figure S2. [file JCMM-27-3363-s002.tif]

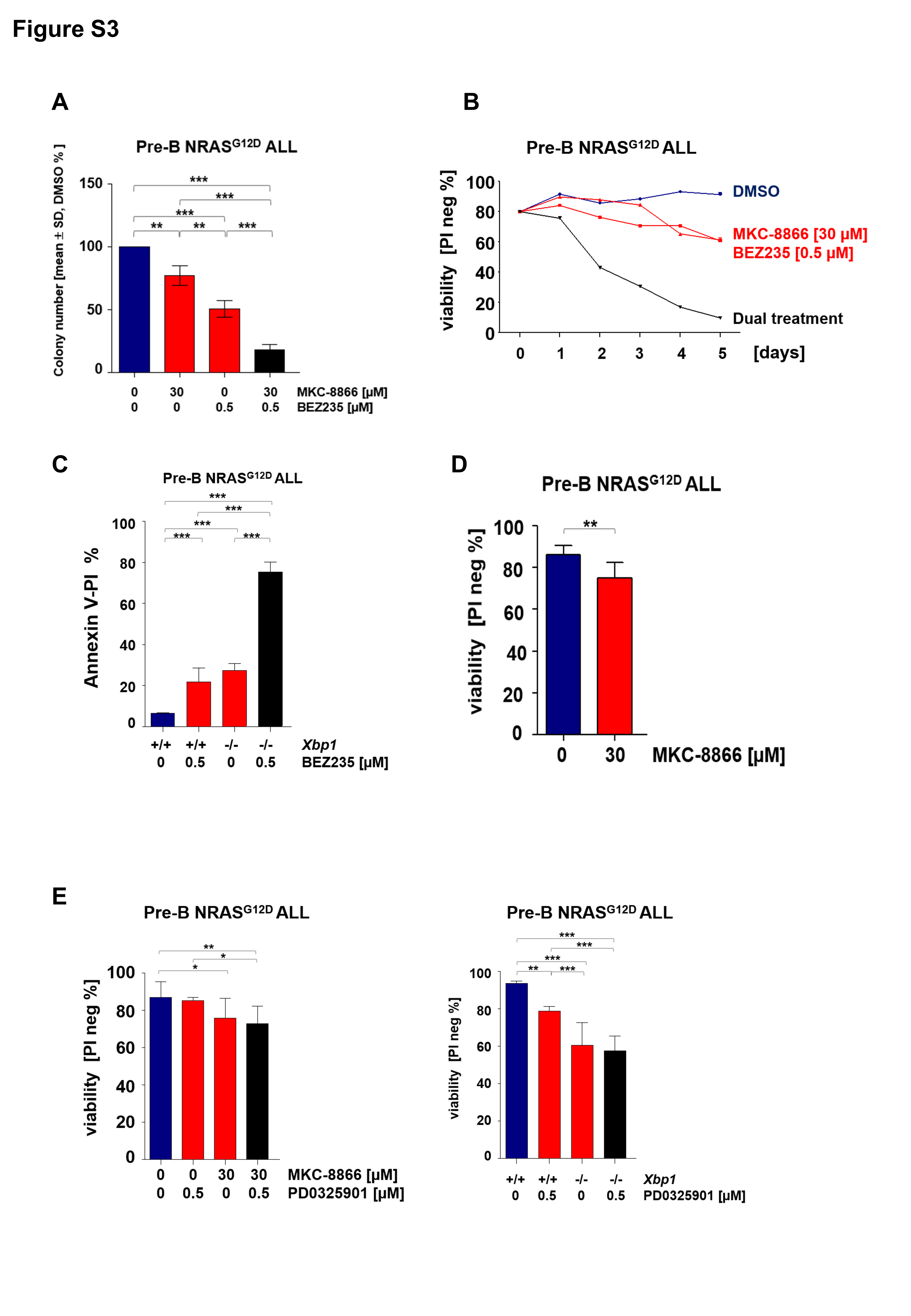

Supplement: Supplementary file 3 — Figure S3. [file JCMM-27-3363-s004.tif]

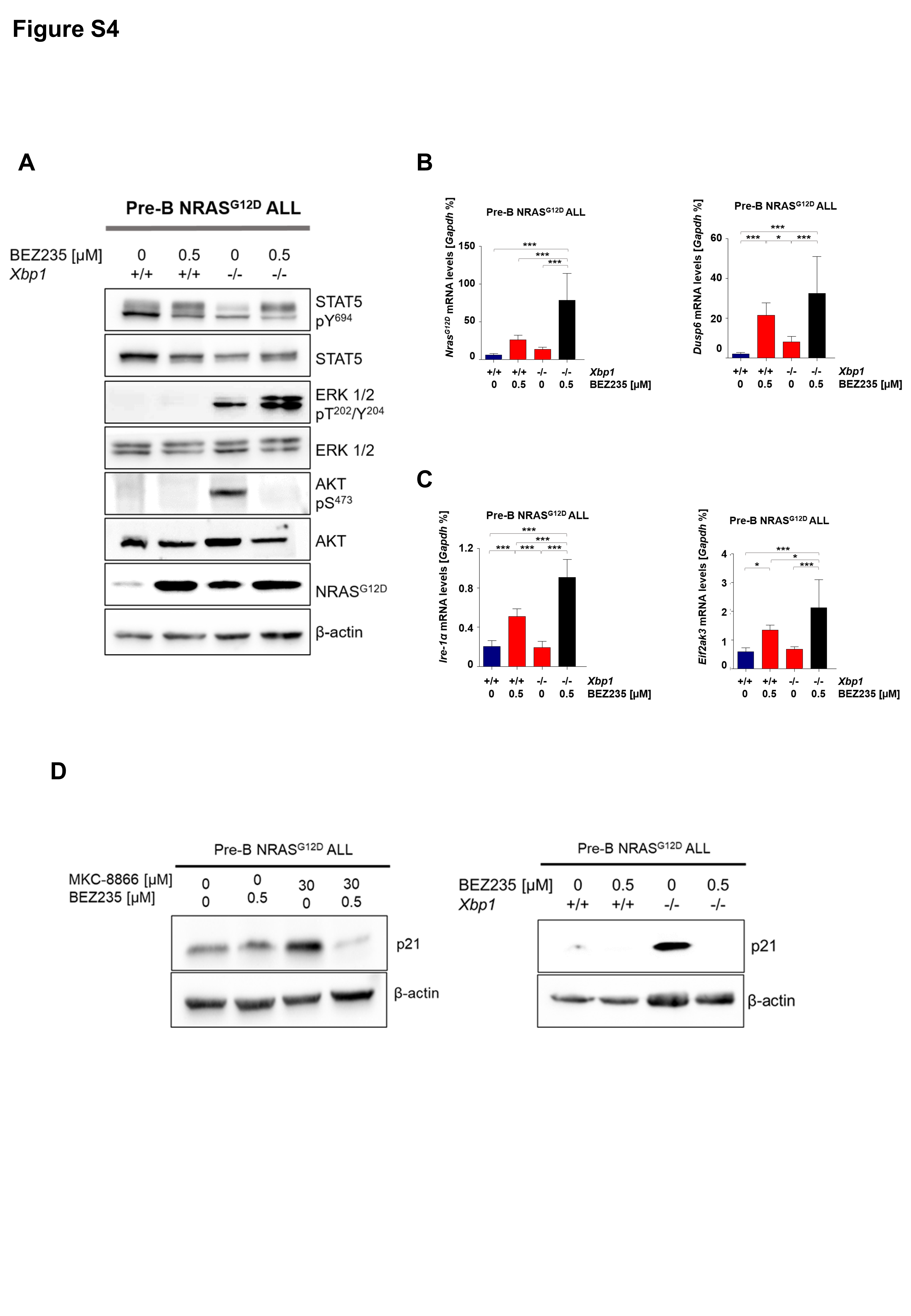

Supplement: Supplementary file 4 — Figure S4. [file JCMM-27-3363-s005.tif]

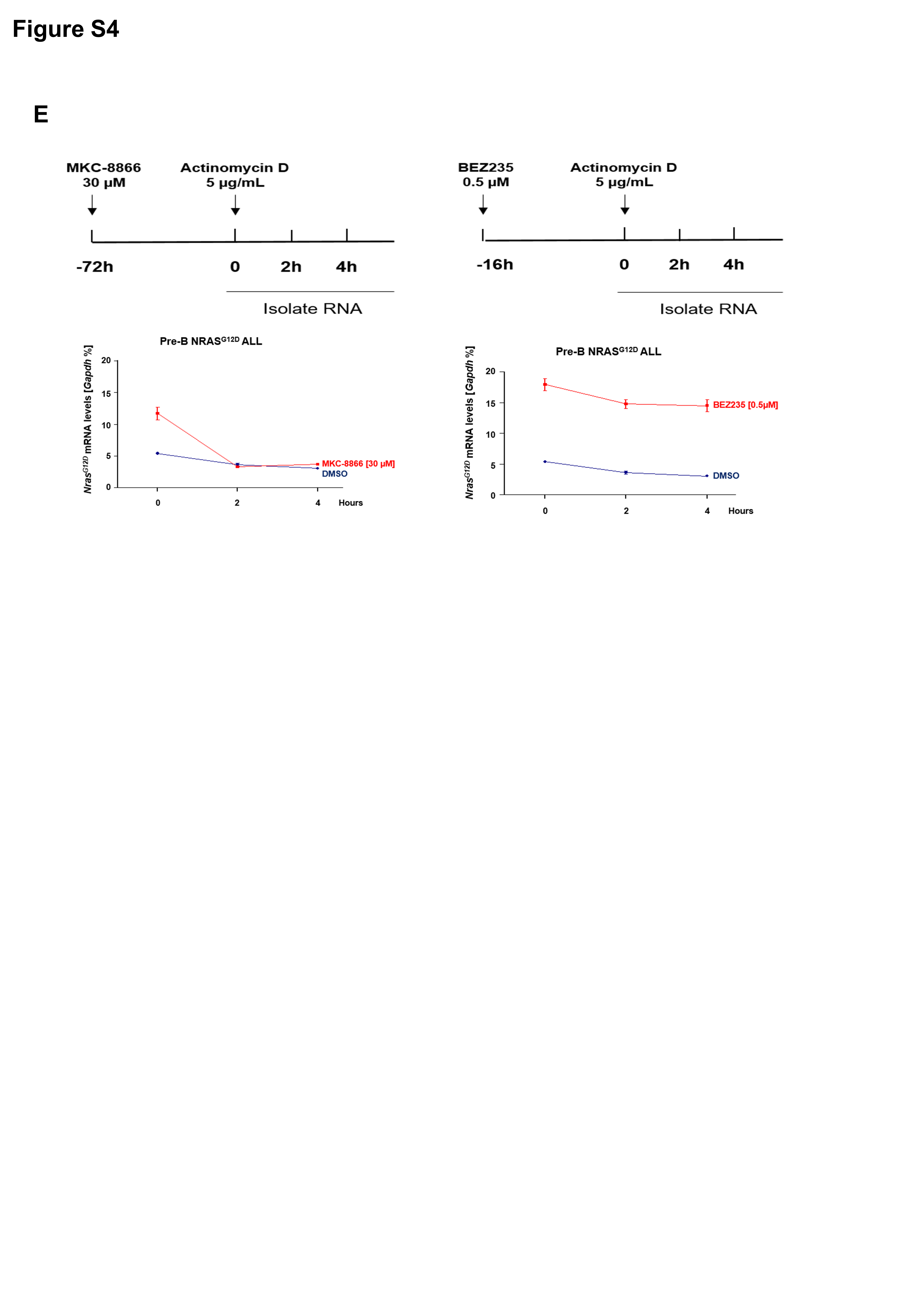

Supplement: Supplementary file 5 — Figure S4. [file JCMM-27-3363-s007.tif]

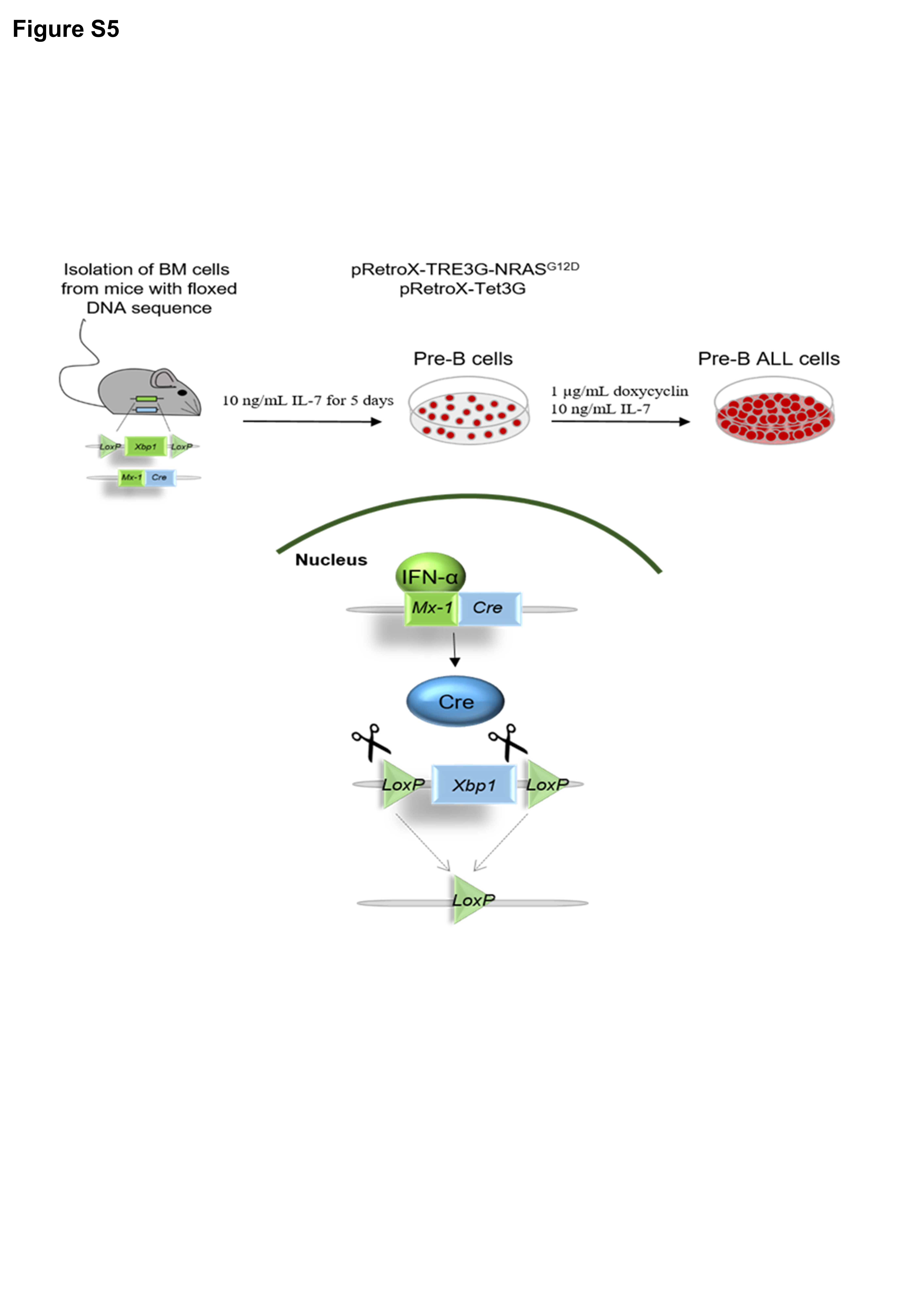

Supplement: Supplementary file 6 — Figure S5. [file JCMM-27-3363-s001.tif]
