# Supplementary material for: XBP1 promotes NRASG12D pre‐B acute lymphoblastic leukaemia through IL‐7 receptor signalling and provides a therapeutic vulnerability for oncogenic RAS
Source: J Cell Mol Med. 2023 Sep 27;27(21):3363–77. doi: 10.1111/jcmm.17904 (PMC10623536; doi:10.1111/jcmm.17904)
Supplement: Supplementary file 7 — Data S1. [file JCMM-27-3363-s006.docx]

**XBP1 promotes NRAS^G12D^ pre-B acute lymphoblastic leukemia through IL-7 receptor signaling and provides a therapeutic vulnerability for oncogenic RAS**

Azam Salimi^1,3,4^, Mirle Schemionek-Reinders^1^, Michael Huber^2^, Margherita Vieri^1^, John B. Patterson^5^ Julia Alten^6^, Tim H. Brümmendorf^1^, Behzad Kharabi Masouleh*^1^, Iris Appelmann^1*+^

- **Figures S1-S5**
- **Supplemental Material and Methods**
- **Tables S1-S8**

**Supplementary figure legends**

**
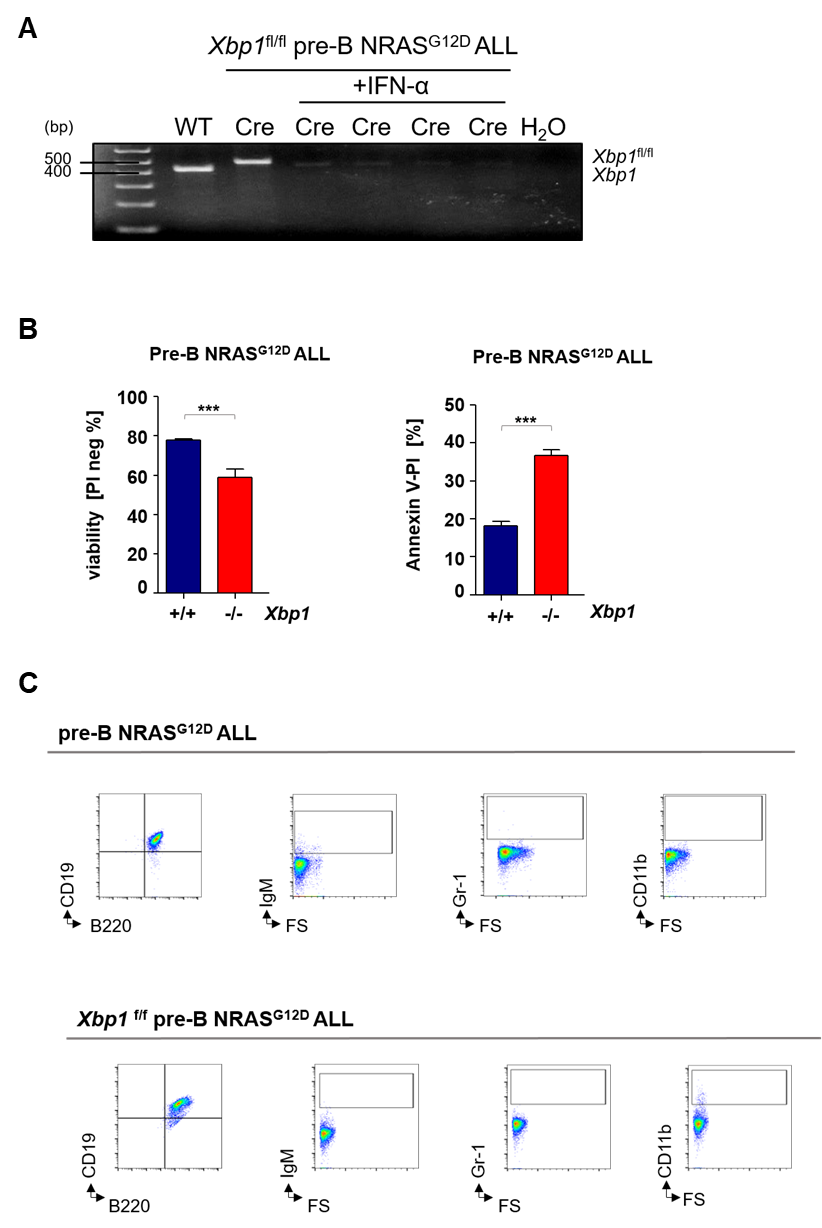
**

**Figure S1. Genetic ablation of *Xbp1* induces cell death in pre-B NRAS^G12D^ ALL cells.** A) Genetic deletion of *Xbp1* was verified using specific primers for *loxP* sites flanking the *Xbp1^fl/fl^* by PCR. B) Cell viability and apoptosis were analyzed in pre-B NRAS^G12D^ ALL cells with EV or Cre after 72 h with 1 µM 4-OHT treatment using PI staining and Annexin-V/PI staining, respectively, n=2. P value was calculated by t-test analysis. C) FACS analysis of B cell lineage and myeloid progenitor surface markers upon loss of *Xbp1* in pre-B NRAS^G12D^ ALL cells.

**
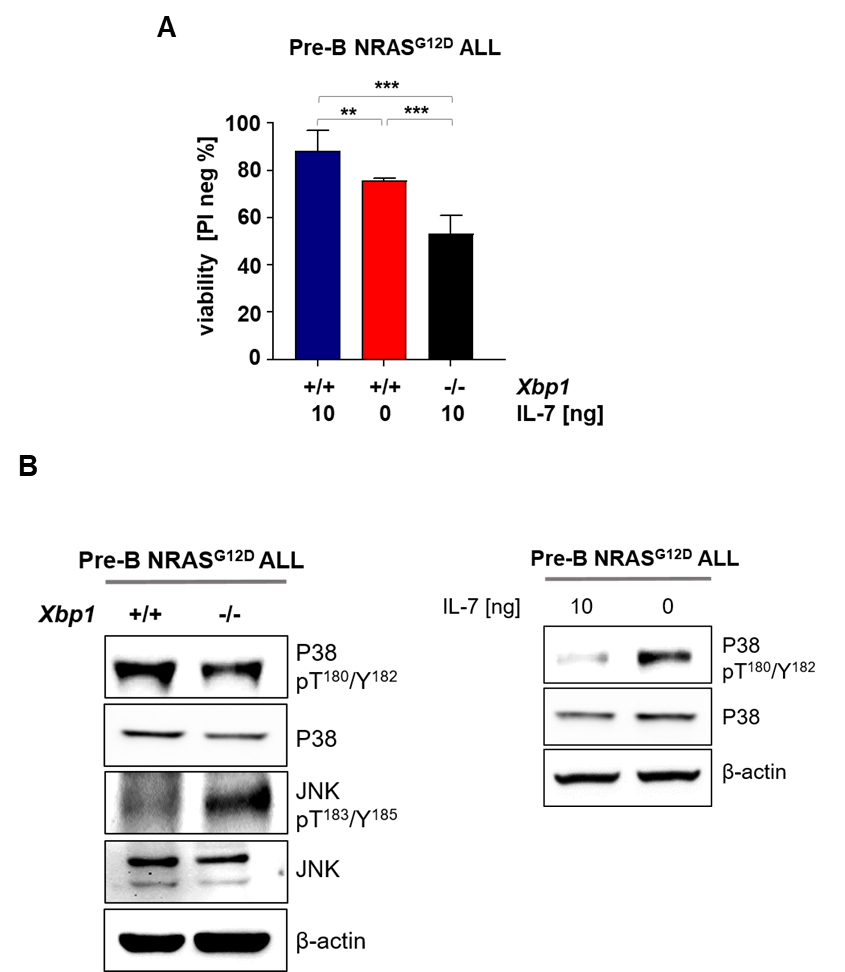
**

**Figure S2. Targeting IL-7R signaling activates stress response kinases.** A) Pre-B NRAS^G12D^ ALL cells were incubated in the presence of IL-7 compared to cells after IL-7 starvation for 24 h. Cell viability was measured by PI staining, n=3. P values were calculated by Student’s t-test. B) Protein levels of total and phosphorylated P38-T^180^/Y^182^ and JNK-T^183^/Y^185^ and beta-actin as loading control were analyzed by western blot in pre-B NRAS^G12D^ ALL cells either in the absence of IL-7 or upon loss of *Xbp1*, n=3.

**
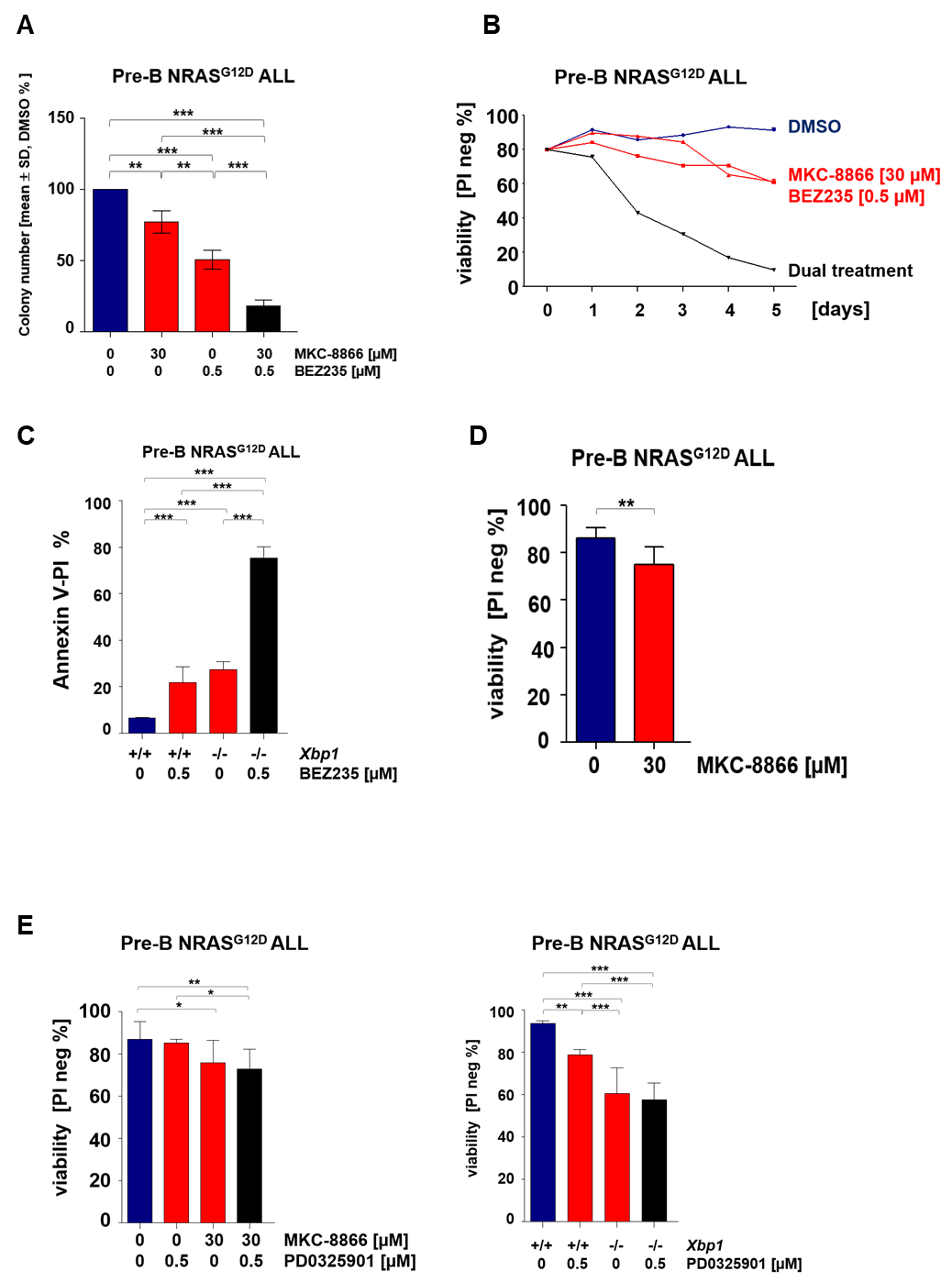
**

**Figure S3. Pharmacological inhibition of XBP1 activation sensitizes RAS-mutated ALL cells to dual inhibitor of the PI3K/mTOR pathway.** Pre-B NRAS^G12D^ ALL cells were treated with 30 µM IRE1α inhibitor MKC-8866 alone or combined either with 0.5 µM MEK inhibitor PD0325901 or 0.5 µM PI3K/mTOR inhibitor BEZ235. A) Colony-forming assay using 10 000 Pre-B NRAS^G12D^ ALL cells in CFU culture medium. Error bars represent mean ± S.D. for each group of treatment, n=3. B) Cell viability of pre-B NRAS^G12D^ ALL cells upon treatment with MKC-8866 combined with BEZ235 over five days, n=3. C) Apoptotic fraction was measured by Annexin-V/PI staining in pre-B NRAS^G12D^ ALL cells upon genetic ablation of *Xbp1* treated with BEZ235 after 72 h, n=2. D) Cell viability were assessed by PI staining after 72 h with MKC-8866 treatment, n=3. P value was calculated by t-test analysis. E) Cell viability in pre-B NRAS^G12D^ ALL cells treated with MKC-8866 and in combination with PD0325901 or upon genetic loss of *Xbp1* treated with PD0325901 after five days using PI staining, n=3. P value was calculated by one-way analysis of variance (ANOVA). C) P value was calculated by one-way analysis of variance (ANOVA).


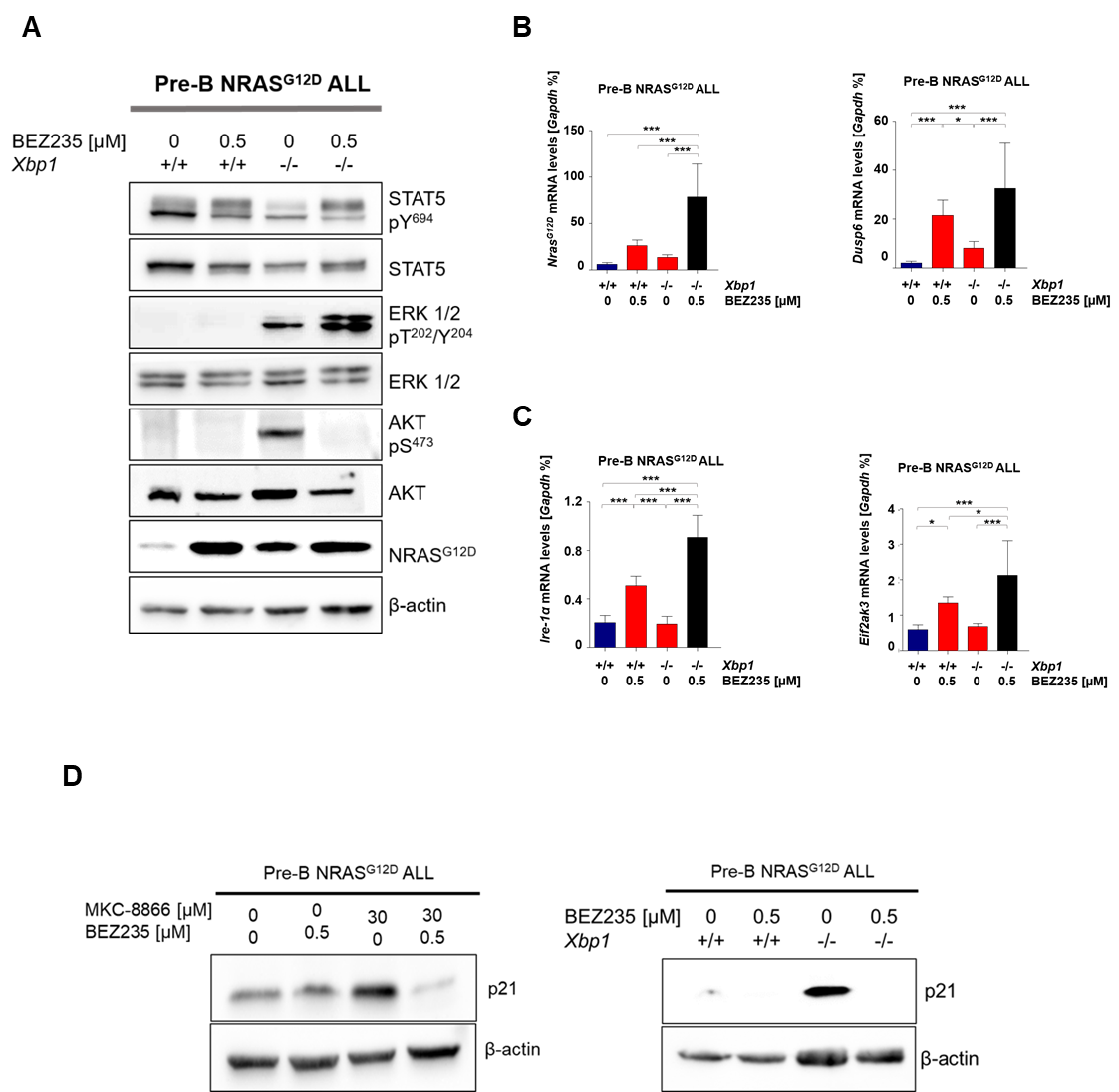


**
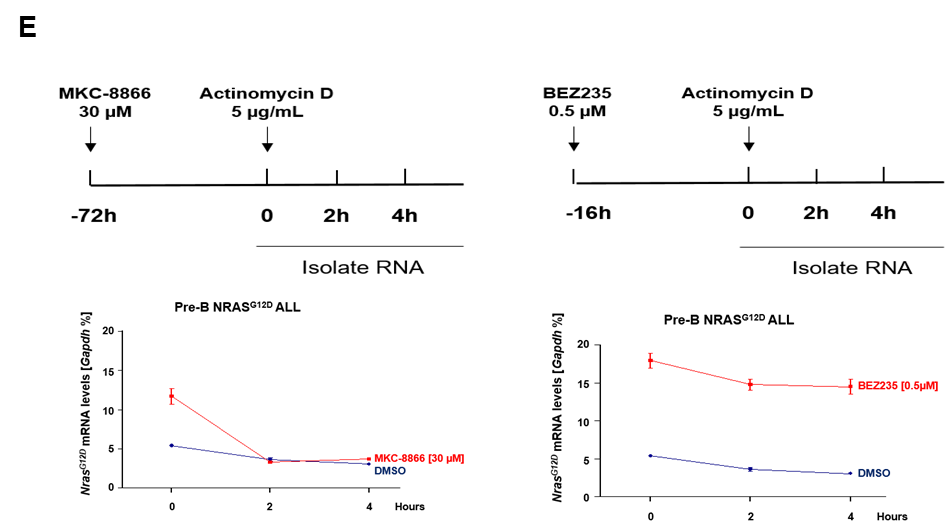
**

**Figure S4. Dual inhibitor of the PI3K/mTOR, BEZ235 upon genetic loss of *Xbp1* fully blocks IL-7R signaling and causes an aberrant Ras-Erk signaling.** Pre-B NRAS^G12D^ ALL cells in absence of *Xbp1* treated with 0.5 µM PI3K/mTOR inhibitor BEZ235 for 16 h. A) Western blot analysis for NRAS^G12D^, STAT5, ERK1/2, AKT, beta-actin as loading control and phosphorylated levels of STAT5-Y^694^, ERK-T^202^/Y^204^ and AKT-S^473^, n=3. B), C) *Nras^G12D^*, *Dusp6*, *Ire1α* and *Eif2ak3* mRNA levels were studied by RT-qPCR, n=3. P value was calculated by one-way analysis of variance (ANOVA). D) Western blot analysis for p21 and beta-actin as loading control after 16 h treatment. E) Pre-B NRAS^G12D^ ALL cells were pretreated as described above. Total RNA was extracted at sequential time points (0-4 h) and mRNA levels of Nras^G12D^ was measured by RT-qPCR.

**
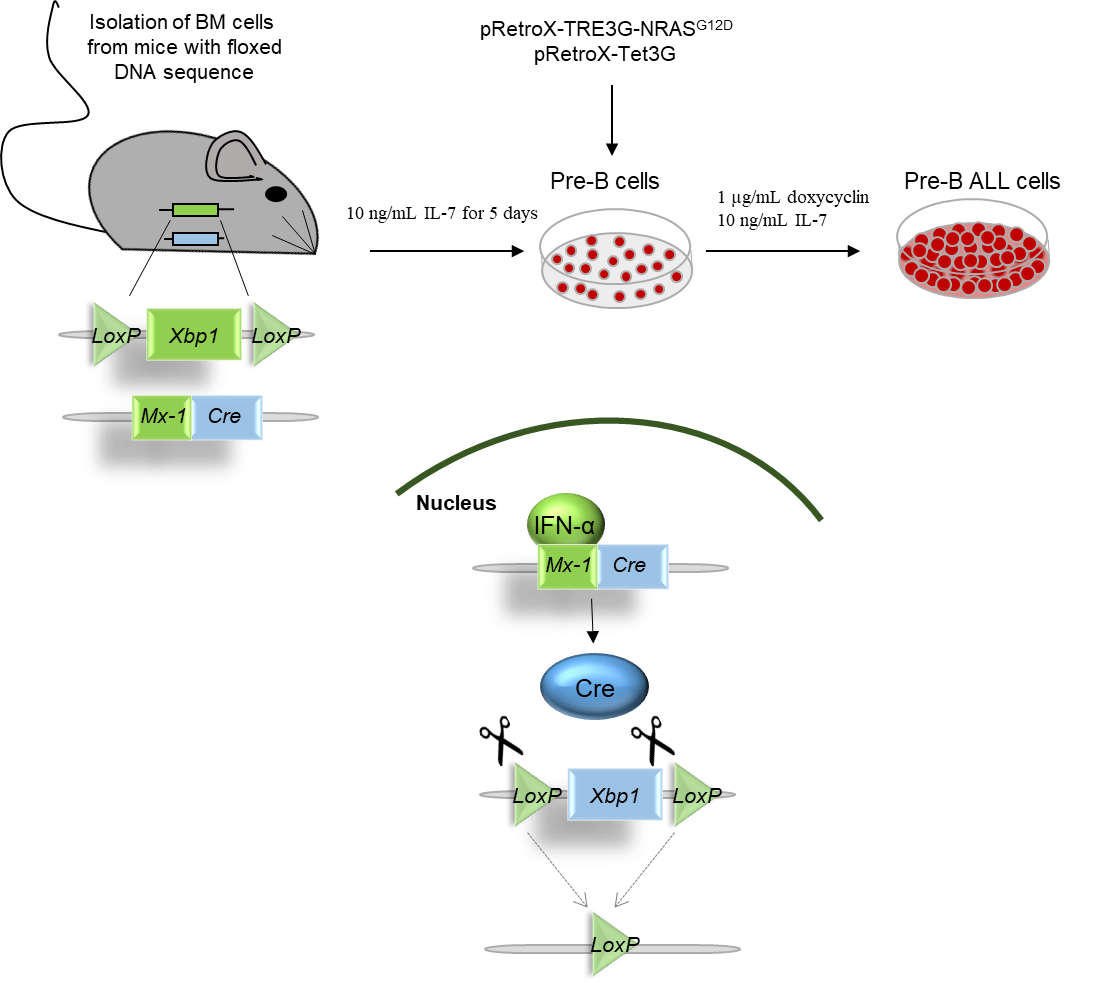
**

**Figure S5. Schematic view of mice model.**

**Supplementary material and methods**

**Mice**

Bone marrow cells were isolated from femurs and tibiae of 6-8 weeks old mice (table S1). The bone marrow cells were flushed using a syringe with a 23-gauge needle containing IMDM supplemented with 20% FBS, 100 IU/mL penicillin, 100 μg/mL streptomycin and 50 μM 2-mercaptoethanol. Afterwards, bone marrow cells were filtered through a 70 µm nylon cell strainer and subjected to red blood depletion using ACK (Ammonium-Chloride-Potassium) buffer containing 150mM NH_4_Cl, 10mM KHCO_3,_ and 0.1 mM Na_2_EDTA at pH 7.4. The bone marrow cells were grown in IMDM with GlutaMAX containing 20% FBS, 100 IU/mL penicillin, 100 μg/mL streptomycin and 50 μM 2-mercaptoethanol supplemented with 10 ng/mL of IL-7 up to five days to establish pre-B cell phenotype. Bone marrow cells from murine *Xbp1*^fl/fl^ were isolated and cultured in Iscove’s Modified Dulbecco’s Medium (IMDM) (Thermo Fisher®) with GlutaMAX containing 20% FBS (Pan Biotech®), 100 IU/mL penicillin, 100 μg/mL streptomycin (life technologies®) and 50 μM 2-mercaptoethanol (Gibco®, Thermo Fisher®), supplemented with 10 ng/mL of Interleukin-7 (Peprotech®). The IL-7-dependent murine *Xbp1*^fl/fl^ pre-B cells were then retrovirally transduced with a Tet-On inducible NRAS^G12D^ vector.

**Retroviral transduction**

Retroviral constructs were transfected using calcium phosphate precipitation (table S2) in platinum-E (Plat-E) cell lines as a retroviral packaging cell. Plat-E cells are grown in Dulbecco's modified eagle medium (DMEM) (Invitrogen^®^) with GlutaMAX containing 10% FBS, 1 μg/mL puromycin, 10 μg/mL blasticidin, 100 IU/mL penicillin and 100 μg/mL streptomycin. The retrovirus supernatants containing NRAS^G12D^ and Tet-On were collected and transduced to *Xbp1^f/f^* pre-B cells or *Mx1-Cre; Xbp1^f/f^* pre-B cells using RetroNectin (Takara) reagents according to the instruction of the manufacture. Subsequently, cells were incubated in the presence of 1 ug/mL doxycycline (Sigma Aldrich) to induce the expression of NRAS^G12D^. *Xbp1^f/f^* pre-B NRAS^G12D^ ALL cells were transduced additionally with inducible-Cre and empty vector (EV) as described above and further GFP positive cells were sorted which performed by the Flow Cytometry Facility, a core facility of the interdisciplinary center for clinical research (IZKF) Aachen within the faculty of medicine at RWTH Aachen university.

**Inducible deletion of the *Xbp1* floxed gene**

In order to induce deletion of the *Xbp1* floxed gene in *Xbp1^f/f^* pre-B NRAS^G12D^ ALL cells, we treated cells with 1 µM of 4-hydroxy tamoxifen (4-OHT) which mediate excision of *loxP* sites flanking the Cre-ER^T2^ *Xbp1^f/f^*. In our second conditional knockout mouse model, *Mx1-Cre; Xbp1^f/f^* NRAS^G12D^ ALL cells treated with 250 U/mL interferon alpha (IFNα) to induce expression of cre as recombinase enzyme to drives deletion of *Xbp1* flanked by *loxP* sites (figure S5). Genetic deletion of Xbp1 was confirmed using the primers listed in table S3.

***In vitro* colony-forming assay**

Mononuclear cells (MNCs) from these samples were extracted by Ficoll® paque and were cultured in Iscove´s Modified Dulbecco´s Medium (IMDM) with GlutaMAX containing 20% fetal bovine serum (FBS, Gibco®), 100 IU/mL penicillin and 100 μg/mL streptomycin (Gibco) were supplemented with 2 mM l-glutamine, 10^−4^ M 2-mercaptoethanol, 10 ng/mL rhIL-7 (peprotech®), in an incubator with a humidified atmosphere of 5% CO2 at 37°C. Cells were treated with the inhibitors (listed in table S7) at specific time points as mentioned in figure legends. For the human or murine CFU assays, 80% methylcellulose without cytokines (Methocult, H4230, M3231, Stem Cell Technologies®, Vancouver, Canada), 20% IMDM, 10^−4^ M 2-mercaptoethanol, 2mM l-glutamine were supplemented with 20 ng/mL rhIL-7, and 1% penicillin/streptomycin. 10 000 BM MNCs were plated in 1 mL of CFU culture medium at 37 °C with 5% CO_2_ for 7-14 days and colonies were counted using inverted light microscopy.

**Flow Cytometry**

To test cell viability, we utilized propidium iodide (PI) staining (Sigma Aldrich®). The apoptotic cell fraction was assessed by Annexin-V/PI staining (BD Bioscience®) with Annexin-V^+^/PI^+^ cells representing late apoptosis. Cell cycle was analyzed by PI staining followed by the quantification of DNA content at the interphase of the cell cycle (G_0/1_, S and G_2/M_) (31). For cellular phenotyping, we measured expression levels of cell surface markers using conjugated murine antibodies (Abs) listed in table S4 by flow cytometry.

**Western Blot**

Cell lysates were obtained using RIPA buffer (radioimmunoprecipitation assay buffer, 150 mM sodium chloride, 1.0% Triton X-100, 0.5% sodium deoxycholate, 0.1% SDS sodium dodecyl sulfate, 50 mM Tris, pH 8.0), supplemented with a protease inhibitor cocktail (0.2 µM of aprotinin, 2 mM AEBSF, 1 µM leupeptin, 10 µM pepstatin A; 130 µM bestatin, 1 µM phosphoramidon, 14 µM E-64, Sigma Aldrich®), and phosphatase inhibitors contains 20 mM sodium orthovanadate (Sigma Aldrich®) and sodium fluoride 50 mM (Roche®). The protein extracts were loaded on pre-cast gels (Bio-Rad®) and were then transferred to a PVDF membrane (Amersham^TM^ Hybond^TM^) and subsequently detected using PCA-ECL solution (100 mM Tris-HCL, pH 8.8, 2.5 mM luminol, 0.198 mM p-coumaric acid and 0.2% v/v hydrogen peroxide (Sigma Aldrich^®^). Finally, blots were visualized by Fusion SL imaging system (Vilber®). Antibodies used in this study were listed in table S5.

**Quantitative real-time PCR**

Total RNA was isolated using a RNeasy plus kit (Qiagen®) according to the manufacturer’s protocol. cDNA was synthesized using an M-MLV Reverse Transcriptase kit (Invitrogen®) and amplified using the iTaq Universal SYBR Green supermix (Bio-Rad®). The reactions were performed by ABI7500 fast real-time PCR system (ThermoFisher Scientific®). Relative quantification of target gene expression was normalized to *Gapdh* applying the comparative threshold cycle. Triplicates of quantitative data are presented as mean ± SD. A list of the primer sequences used is provided in table S6.

**Table S1: The mouse strains used in this study.**

| Mouse strain | Source | | | Purpose |
| --- | --- | --- | --- | --- |
| *Xbp1*^fl/fl^ | Laurie H. Glimcher, Cornell Medical School | | | Genetic loss-of-function experiments |
| *Mx1-Cre* | | Jackson Laboratories | Inducible deletion of floxed genes | |

**Table S2: Retroviral vectors used in this study.**

| Construct | Inducible overexpression |
| --- | --- |
| pRetroX-TRE3G-NRAS^G12D^-Puro | NRAS^G12D^; Puromycin resistance |
| pRetroX-Tet3G-Neo | Tet-On; Neomycin resistance |
| MSCV-Cre-ER^T2^-IRES-GFP | Cre-ERT2; GFP |
| MSCV- ER^T2^-IRES-GFP | GFP |

**Table S3:** **Oligos used in this study.**

| Oligo name | Sequences |
| --- | --- |
| *Xbp1-F* | 5’-TTTGGCTTGGGGAGGGACA-3’ |
| *Xbp1-R* | 5’-AGCAGTCTGCGCTGCTACTCT-3’ |
| *Mx1-Cre-F* | 5’-CATTTGGGCCAGCTAAACAT-3’ |
| *Mx1-Cre-R* | 5’-TAAGCAATCCCCAGAAATGC-3’ |

**Table S4. The murine flow cytometry antibodies used in this study.**

| Surface antigen | | Clone ID | Source |
| --- | --- | --- | --- |
| CD19-PE | | eBio1D3 | BD Biosciences |
| CD45R/B220-FITC | | RA3-6B2 | BD Biosciences |
| Ly-6G/Gr-1-PE/Cy5 | | RB6-8C5 | Biolegend |
| CD11b/ Mac-1-PE/Cy5 | M1/70 | | Biolegend |
| IgM-PE | | R6-60.2 | BD Biosciences |
| Annexin-V-APC | |  | BD Biosciences |

**Table S5:** **Antibodies used for Western blotting in this study.**

| Antigen | Clone ID | Source |
| --- | --- | --- |
| Beta Actin | Polyclonal (ab8227) | Abcam |
| p21 | EPR3993 | Abcam |
| p27 | D69C12 | Cell Signaling Technology |
| MKP-3 (DUSP6) | G-4 | Santa Cruz Biotechnology |
| Phospho-JAK1 | Polyclonal (44-422G) | Invitrogen |
| JAK1 | 73/JAK1 | BD Bioscience |
| Phospho-AKT | D9E | Cell Signaling Technology |
| AKT | Polyclonal (9272) | Cell Signaling Technology |
| Phospho-ERK1/2 | 20G11 | Cell Signaling Technology |
| ERK1/2 | Polyclonal (9102) | Cell Signaling Technology |
| Phospho-STAT5 | Polyclonal (9351) | Cell Signaling Technology |
| STAT5 | D206Y | Cell Signaling Technology |
| Phospho-SAPK/JNK | 81E11 | Cell Signaling Technology |
| SAPK/JNK | Polyclonal (9252) | Cell Signaling Technology |
| Phospho-p38 | D3F9 | Cell Signaling Technology |
| p38 | Polyclonal (9212) | Cell Signaling Technology |
| RAS (G12D Mutant) | D8H7 | Cell Signaling Technology |

**Table S6:** **The list of murine primers used for RT-qPCR in this study.**

| Oligo name | Sequences |
| --- | --- |
| *Gapdh-F* | 5’-CATGGCCTTCCGTGTTCCTA-3’ |
| *Gapdh-R* | 5’-CCTGCTTCACCACCTTCTTGAT-3’ |
| *Xbp1s-F* | 5’-GACAGAGAGTCAAACTAACGTGG-3’ |
| *Xbp1s-R* | 5’-GTCCAGCAGGCAAGAAGGT-3’ |
| *Dusp6-F* | 5’-ATAGATACGCTCAGACCCGTG-3’ |
| *Dusp6-R* | 5’-ATCAGCAGAAGCCGTTCGTT-3’ |
| *Etv5-F* | 5’-TCAGTCTGATAACTTGGTGCTTC-3’ |
| *Etv5-R* | 5’-GGCTTCCTATCGTAGGCACAA-3’ |
| *Spry2-F* | 5’-TCCAAGAGATGCCCTTACCCA-3’ |
| *Spry2-R* | 5’-GCAGACCGTGGAGTCTTTCA-3’ |
| *Nras-G12D-F* | 5’-CTGGTGGTGGTTGGAGCAGA-3’ |
| *Nras-G12D-R* | 5’-GGTTTCACCATCTATAACCAC-3’ |
| *Il7r-F* | 5’-GCGGACGATCACTCCTTCTG-3’ |
| *Il7r-R* | 5’-AGCCCCACATATTTGAAATTCCA-3’ |
| *Ern1-F* | 5’-GCAACCATCCTTTTGGCAAAT-3’ |
| *Ern1-R* | 5’-AACAGTCAAGGTTGCAGGCG-3’ |
| *Eif2ak3-F* | 5’-GCGTCGGAGACAGTGTTTG-3’ |
| *Eif2ak3-R* | 5’-CGTCCATCTAAAGTGCTGATGAT-3’ |

**Table S7. The list of therapeutic agents used in this study.**

| Compound | Target | Source |
| --- | --- | --- |
| MKC-8866 | IRE1α RNase | Mankind |
| PD0325901 | MEK1/2 | Selleckchem |
| BEZ235 | PI3K/mTOR | Cellagen Technology |

**Table S8. The list of tumor samples from patients with RAS-mutated ALL.**

| Sample ID | Timepoint |  | Material |
| --- | --- | --- | --- |
| Ki1 | Initial | BM | Cells |
| Ki2 | initial | BM | Cells |
| Ki3 | initial | BM | Cells |
| Ki4 | initial | BM | RNA |
| Ki5 | initial | BM | Cells |
| Ki6 | initial | BM | Cells |
